# CIP2A induces PKM2 tetramer formation and oxidative phosphorylation in non-small cell lung cancer

**DOI:** 10.1038/s41421-023-00633-0

**Published:** 2024-02-06

**Authors:** Li-Jun Liang, Fu-Ying Yang, Di Wang, Yan-Fei Zhang, Hong Yu, Zheng Wang, Bei-Bei Sun, Yu-Tao Liu, Gui-Zhen Wang, Guang-Biao Zhou

**Affiliations:** 1 State Key Laboratory of Molecular Oncology & Department of Medical Oncology, National Cancer Center/National Clinical Research Center for Cancer/Cancer Hospital, Chinese Academy of Medical Sciences and Peking Union Medical College, Beijing, China; 2 Department of Thoracic Surgery, Second Affiliated Hospital, School of Medicine, Zhejiang University, Hangzhou, Zhejiang China; 3 Department of Clinical Laboratory, Peking Union Medical College Hospital, Chinese Academy of Medical Sciences and Peking Union Medical College, Beijing, China; 4 Department of Basic Medicine, Anhui Medical College, Hefei, Anhui China; 5 Department of Pharmacology, University of Texas Health Science at San Antonio, San Antonio, TX USA

**Keywords:** Cancer metabolism, Mechanisms of disease

## Abstract

Tumor cells are usually considered defective in mitochondrial respiration, but human non-small cell lung cancer (NSCLC) tumor tissues are shown to have enhanced glucose oxidation relative to adjacent benign lung. Here, we reported that oncoprotein cancerous inhibitor of protein phosphatase 2A (CIP2A) inhibited glycolysis and promoted oxidative metabolism in NSCLC cells. CIP2A bound to pyruvate kinase M2 (PKM2) and induced the formation of PKM2 tetramer, with serine 287 as a novel phosphorylation site essential for PKM2 dimer-tetramer switching. CIP2A redirected PKM2 to mitochondrion, leading to upregulation of Bcl2 via phosphorylating Bcl2 at threonine 69. Clinically, CIP2A level in tumor tissues was positively correlated with the level of phosphorylated PKM2 S287. CIP2A-targeting compounds synergized with glycolysis inhibitor in suppressing cell proliferation in vitro and in vivo. These results indicated that CIP2A facilitates oxidative phosphorylation by promoting tetrameric PKM2 formation, and targeting CIP2A and glycolysis exhibits therapeutic potentials in NSCLC.

## Introduction

Cancer cells exhibit the Warburg effect (aerobic glycolysis) and are usually considered to be defective in mitochondrial respiration^1,2^. However, recent evidence indicates that cancer cells also retain mitochondrial respiration^2,3^, which enables cell proliferation^3^. By using intraoperative ^13^C-glucose infusions in vivo, human non-small cell lung cancer (NSCLC) tumors are shown to have enhanced glucose oxidation relative to adjacent benign lung^4^. Consistently, murine lung cancer tumor growth requires the electron transport chain to oxidize ubiquinol, while lack of mitochondrial complex III in cancer cells impairs tumor growth^5^. KRAS-driven mouse lung tumors have increased glucose contribution to the tricarboxylic acid (TCA) cycle relative to normal lung tissue, and mitochondrial metabolism of pyruvate is essential for in vivo tumor formation^6^. At cellular level, lung adenocarcinoma cells exhibit significant increases in mitochondrial complex I and complex II respiratory activity^7^; cholangiocarcinoma cancer stem cells (CSCs) have a more efficient respiratory phenotype and depend on mitochondrial oxidative metabolism to maintain stemness^8^, while pancreatic cancer CSCs are dependent on oxidative phosphorylation (OXPHOS) with very limited metabolic plasticity^9^. Some oncoproteins, such as C-JUN, can inhibit glycolysis pathways^10^, while MYC and MCL1 cooperate to maintain CSC resistance to chemotherapy by increasing OXPHOS and reactive oxygen species production in triple-negative breast cancer.^11^ However, the roles and mechanisms of oxidative metabolism in cancer cells remain to be elucidated.

The ability of protein phosphatase 2A (PP2A), a serine/threonine phosphatase that consists of a catalytic C subunit, a structural A subunit and a regulatory/variable B subunit^12^, is inhibited by cancerous inhibitor of PP2A (CIP2A), a multifunctional oncoprotein previously known as KIAA1524 or p90^13–15^. CIP2A interacts with more than 100 proteins (https://thebiogrid.org/121687/summary/homo-sapiens/kiaa1524.html) and is involved in many signaling pathways^16–18^. CIP2A binds and prevents proteolytic degradation of c-Myc, and interacts with DNA topoisomerase II binding protein 1 (TopBP1)^17^ to safeguard chromosome stability^19^ and poises pulverized chromosomes for clustering upon mitotic entry^20^. CIP2A modulates cell cycle progression^17,21^, promotes malignant transformation^14,22,23^, and renders cancer cell drug resistance^24^. CIP2A is overexpressed in > 70% of solid or hematological malignancies and is associated with poor prognosis of the patients^25^. CIP2A deficiency in mice leads to resistance to DMBA (7,12-dimethylbenz[a]anthracene)-induced basal-like breast cancer formation^17^, and inhibition of CIP2A induces cell cycle arrest, promotes apoptosis or cellular senescence, and exhibits therapeutic efficacies in vitro and in vivo^26,27^. Therefore, CIP2A plays important roles in carcinogenesis, and more efforts to dissect its biological functions may help develop CIP2A inhibitors to treat cancers.

Pyruvate kinase M (PKM) is the final rate-limiting enzyme in glycolysis, catalyzing the conversion of phosphoenolpyruvate to pyruvate with concomitant formation of ATP. The *PKM* gene encodes two alternative-splicing products, PKM1 and PKM2, by mutually exclusive use of exons 9 and 10^28^. PKM2 structurally exists as monomer, dimer, or tetramer. The tetrameric PKM2 exerts pyruvate kinase catalytic activity, whereas the nuclear dimeric PKM2 serves as a transcriptional coactivator or protein kinase to promote aerobic glycolysis and tumor progression^29–32^. PKM2 is conformationally coordinated by endogenous metabolic intermediates or cofactor binding-induced posttranslational modification^33–38^. For example, the acetyltransferase p300 directly binds PKM2 and acetylates it at K433, causing its dissociation into dimers by interfering with the binding to its allosteric activator fructose 1,6-bisphosphate (FBP)^39^. Some modification sites (e.g., S37 phosphorylated by epidermal growth factor receptor (EGFR)) located far away from the FBP-binding pocket or dimer–dimer interface, can also impact PKM2 oligomerization through unclear mechanism^40^. In contrast, serine can bind to PKM2 and facilitate tetramer formation to activate PKM2, whereas serine deprivation leads to reduced PKM2 activity in cells^37^. A small molecule succinylaminoimidazolecarboxamide ribose-5′-phosphate (SAICAR) induces PKM2 dimer-tetramer switching and enhances its pyruvate kinase activity^38^. Cancer is addicted to gain-of-function oncoprotein and loss-of-function tumor suppressor signaling pathways, but the roles of oncoproteins and tumor suppressors in regulation of PKM2 dimer-tetramer switching still need to be addressed.

Several reports have demonstrated that CIP2A is associated with metabolic reprogramming. In chronic myeloid leukemia cell line K562, CIP2A augments mitochondrial respiration^41^. In human retinal pigment epithelium RPE1 cells, CIP2A depletion shifts metabolism towards glycolytic pathway, increases the expression of glycolysis-related genes, and downregulates TCA cycle-related genes^42^. However, the mechanisms of action of CIP2A in metabolic reprogramming and its potential interaction with metabolic enzymes need to be further investigated.

In this study, we investigated the roles and mechanisms of action of CIP2A in metabolic reprogramming, and found that CIP2A promoted oxidative metabolism in NSCLC cells through induction of PKM2 oligomerization, with S287 as key amino acid in determining dimer-tetramer switching. CIP2A upregulated Bcl2 via PKM2-mediated phosphorylation of Bcl2 at threonine (T) 69. CIP2A inhibitors in combination with glycolysis inhibitor exerted enhanced inhibitory effects on NSCLC cells in vitro and in vivo. Our results revealed novel functions of CIP2A in regulation of PKM2 oligomerization and provided therapeutic potentials for NSCLC.

## Results

### CIP2A deficiency activates glycolytic metabolism in NSCLC cells

**Fig. 1:**
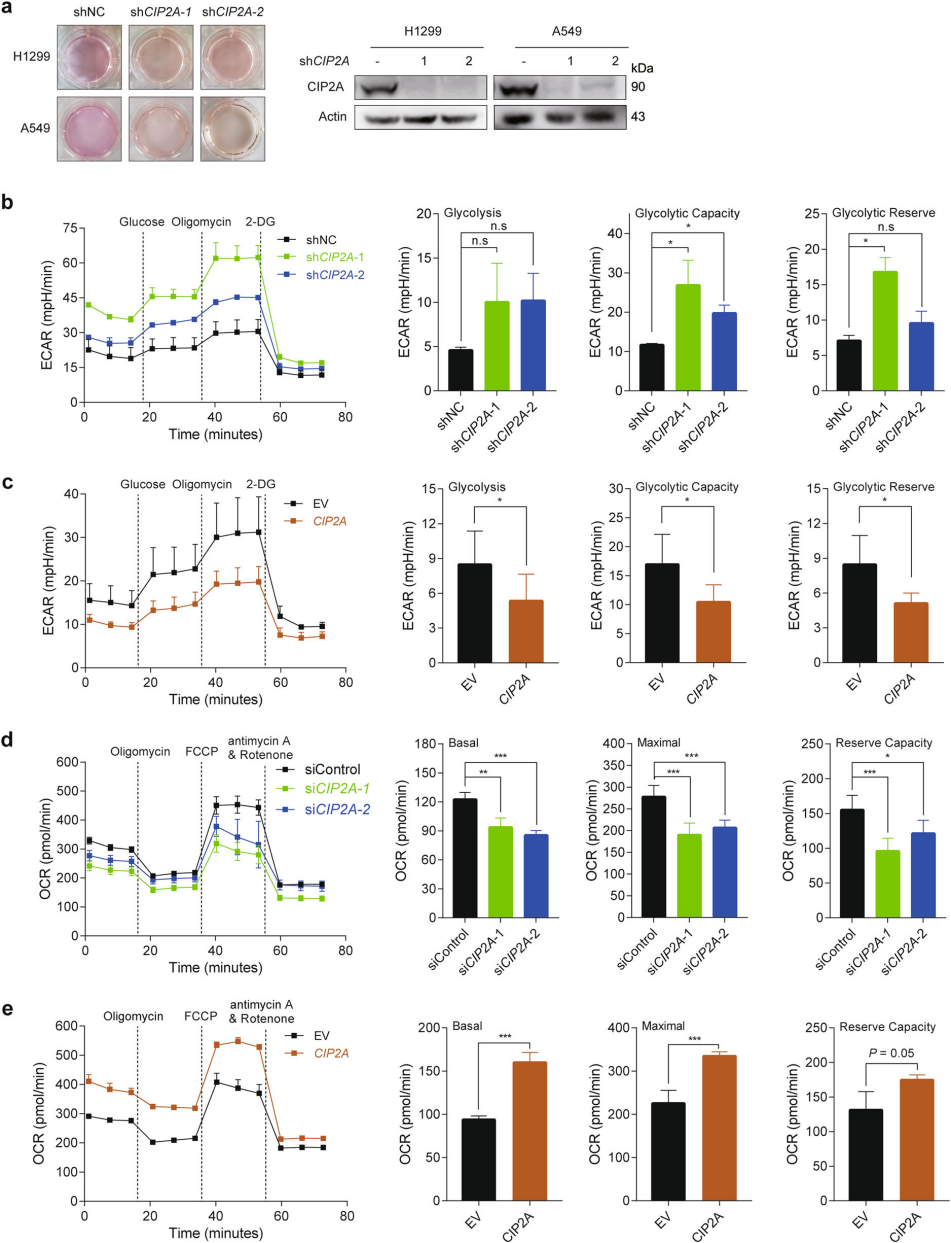
CIP2A modulates ECAR and OCR in NSCLC cells. **a** Representative bright-field images of culture media of H1299 and A549 cells that were transfected with si*CIP2A*. **b**, **c** ECAR of H1299 cells that were transfected with si*CIP2A* (**b**) or pCDH-CMV-*CIP2A* (**c**), measured with a Seahorse XFe96 analyzer. **d**, **e** OCR of H1299 cells that were transfected with si*CIP2A* (**d**) or pCDH-CMV-*CIP2A* (**e**), measured with a Seahorse XFe96 analyzer. Two-sided unpaired Student’s *t-*test; *P < 0.05, **P < 0.01, ***P < 0.001; n.s. not significant.

Our previous work showed that CIP2A was elevated and had an important role in human NSCLC^27,43,44^. During daily culture of NSCLC cell lines, we observed that the medium of CIP2A-knockdown cells (by transfection of sh*CIP2A*; Fig. 1a and Supplementary Fig. S1a, b) turned orange much more rapidly than that of control cells, even at the same confluence. This observation indicated an increased production of acidic metabolites in CIP2A-knockdown cells, which prompted us to investigate the role of CIP2A in glucose metabolism. We applied XFe Seahorse energy metabolic stress assays to determine the impact of CIP2A on glucose metabolism, and the rate of glycolysis was assessed by extracellular acidification rate (ECAR). As anticipated, the glycolysis level, maximum glycolytic capacity, and glycolytic reserve were significantly increased in CIP2A-knockdown H1299 cells (Fig. 1b), whereas the reverse was observed in CIP2A-overexpressing cells (Fig. 1c). In CIP2A-knockdown H1299 cells, significant reductions in the basal mitochondrial respiration level, maximum rate of OXPHOS and reserve capacity of OXPHOS, as measured by oxygen consumption rate (OCR), were observed (Fig. 1d). In contract, significant increases in the basal mitochondrial respiration level, maximum rate of OXPHOS and reserve capacity of OXPHOS, were seen in H1299 cells that were transfected with pCDH-CMV-*CIP2A* (Fig. 1e).

### Identification of PKM2 as an endogenous CIP2A-binding protein

**Fig. 2:**
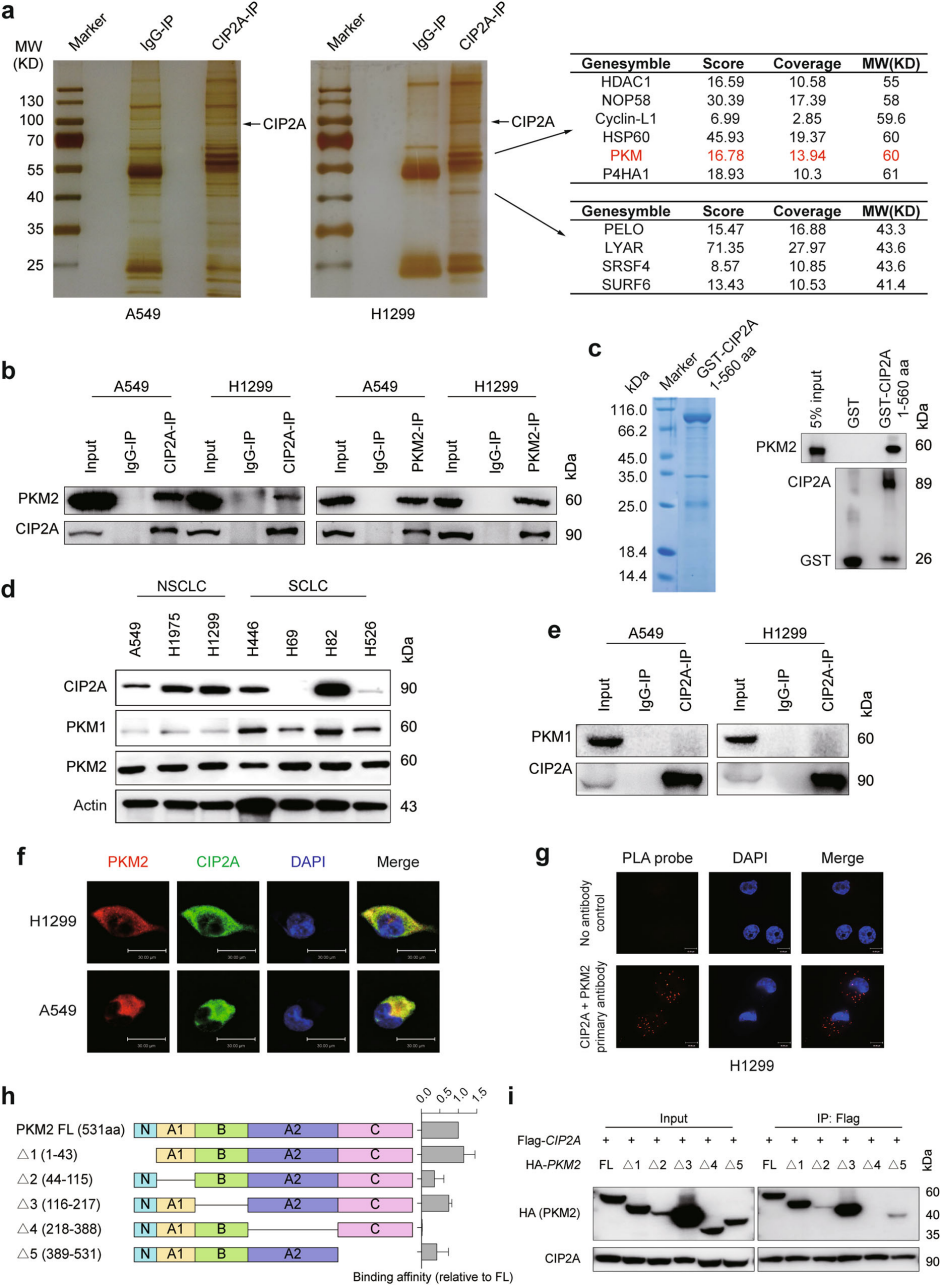
CIP2A physically interacts with PKM2. **a** MS analysis of CIP2A-interacting proteins. Cellular extracts were prepared from A549 and H1299 cells, followed by immunoprecipitation (IP) with an anti-CIP2A antibody. The distinct protein bands on silver-stained SDS-PAGE gels were retrieved and analyzed by MS. **b** IP and immunoblotting assays using the indicated antibodies and cell lysates. **c** GST pull-down and western blot assays. Left, Coomassie blue staining of GST-CIP2A (1–560 aa). Right, GST or GST-CIP2A (1–560 aa) was incubated with His-PKM2 in vitro and the samples were analyzed by western blotting using antibodies against PKM2 and GST. **d** The expression of CIP2A, PKM1 and PKM2 was detected by western blot in the indicated cells. **e** Interaction between endogenous PKM1 and CIP2A proteins was detected by co-IP assay followed by western blot using the anti-CIP2A antibody in A549 and H1299 cells. **f** IF assays of H1299 and A549 cells using antibodies against PKM2 (red) and CIP2A (green), and DAPI to stain nuclei (blue). Scale bars, 30 μm. **g** Representative images of PLA using the Duolink in situ PLA Probe and anti-CIP2A and anti-PKM2 antibodies in H1299 cells. No primary antibodies were used for negative controls. Positive PLA signals indicating the CIP2A–PKM2 complex are shown as red puncta. Scale bars, 10 μm. **h** Schematic diagram representing PKM2 protein fragments used to identify the CIP2A binding region. **i** HA-tagged *PKM2* mutants and Flag-tagged *CIP2A* were co-transfected into 293T cells, which were used for co-IP and subsequent western blotting.

To gain mechanistic insights, endogenous CIP2A was immunoprecipitated from extracts of A549 and H1299 cells, the eluates were resolved by SDS-PAGE, the gels were silver-stained and several major bands not seen in the control lane were subjected to mass spectrometry (MS) to identify CIP2A-interacting proteins. Interestingly, PKM was discovered as a CIP2A-binding protein (Fig. 2a). To verify this interaction, co-immunoprecipitation (co-IP) analyses were performed using cell lysates of A549 and H1299 cells, and we found that CIP2A could pull down PKM2 and vice versa (Fig. 2b). Additionally, GST-CIP2A could pull down PKM2 from H1299 cell lysates (Fig. 2c and Supplementary Fig. S2), suggesting that CIP2A could directly interact with PKM2. We tested whether CIP2A interacts with PKM1, despite the expression of PKM1 was much lower in NSCLC cells than in small cell lung cancer (SCLC) cells (Fig. 2d), which was consistent with a previous study^45^. We found that PKM1 was unable to interact with CIP2A (Fig. 2e). To further confirm the interaction between CIP2A and PKM2, we detected the colocalization of CIP2A and PKM2 by immunofluorescence (IF) staining in A549 and H1299 cells. The results indicated that both CIP2A and PKM2 were primarily localized in the cytoplasm and that CIP2A colocalized with PKM2 (Fig. 2f and Supplementary Fig. S1b). This observation was confirmed by Duolink proximity ligation assay (PLA), a technology that permits detection of protein–protein interactions in situ (at distances < 40 nm) at endogenous protein levels (Fig. 2g). We next determined the interacting domain for the PKM2–CIP2A interaction. As shown in Fig. 2h, a series of PKM2 truncated mutants fused to the HA tag were generated to test the binding affinity with Flag-tagged CIP2A in 293T cells by co-IP. We showed that deletion of the A2 domain (amino acids between 218 and 388) abrogated the interaction between PKM2 and CIP2A, whereas the other fragments of PKM2 retained the ability to bind CIP2A with various affinities (Fig. 2i). These results indicated that CIP2A could bind PKM2 via the A2 domain.

### CIP2A stabilizes PKM2 oligomerization

**Fig. 3:**
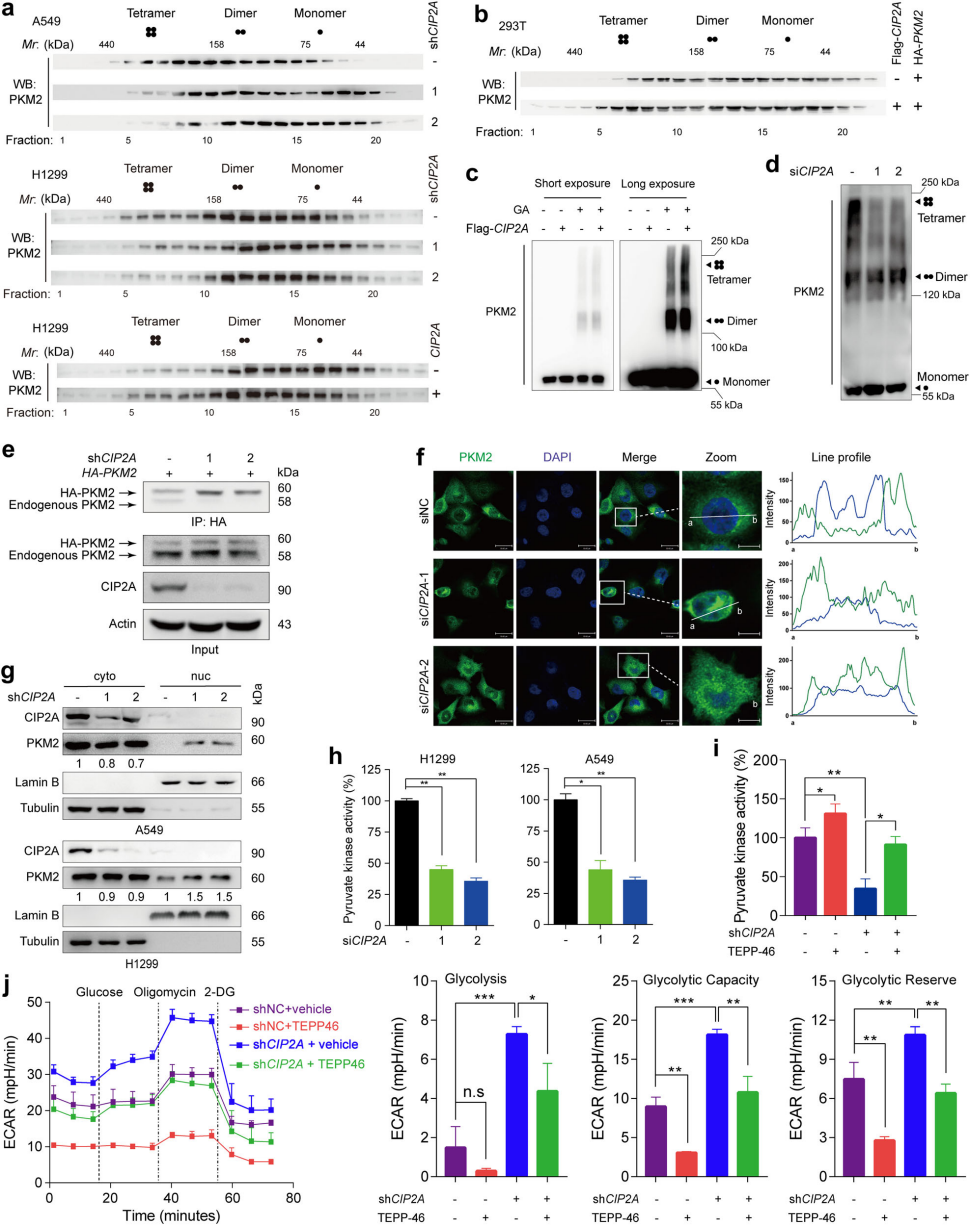
CIP2A inhibits glycolysis by stabilizing oligomerization, upregulating the activity and hindering the nuclear translocation of PKM2. **a** CIP2A knockdown shifts PKM2 to lower-molecular-weight fractions. Cell lysates from A549 cells that were transfected with sh*CIP2A* (upper panel), or H1299 cells that were transfected with sh*CIP2A* (middle panel) or pCDH-*CIP2A* (lower panel), were separated by size exclusion chromatography and analyzed by western blotting. The fraction number and relative molecular weight (Mr) are indicated. **b** CIP2A shifts PKM2 to higher-molecular-weight fractions. The indicated plasmids were co-transfected into 293T cells, and cell lysates were separated by gel filtration, followed by western blot analysis. The fraction number and Mr are indicated. **c** Cell lysates from H1299 cells transiently expressing Flag-*CIP2A* were incubated with or without the crosslinking reagent of 0.01% glutaraldehyde and then subjected to western blot assay using an anti-PKM2 antibody. GA, glutaraldehyde. **d** H1299 cells were transfected with si*CIP2A* for 36 h, lysed, and the cell lysates were crosslinked with 0.01% glutaraldehyde at 37 °C for 9 min. Immunoblot analyses were performed with an anti-PKM2 antibody. **e** Silencing of CIP2A reduces the intermolecular interactions with PKM2. CIP2A-knockdown H1299 cells were transfected with HA-*PKM2*, lysed, and subjected to co-IP assays to examine the interactions of endogenous PKM2 with HA-PKM2. **f** Subcellular localization of PKM2 in H1299 cells transfected with si*CIP2A* was examined by IF assay. Cells were immunostained with an anti-PKM2 antibody (green), and DAPI (blue) was used to mark the nucleus. The line profiles of PKM2 and DAPI signals were determined with ZEN 2011 (Carl Zeiss) software. Scale bars, 30 μm for unmagnified image and 10 μm for magnified image. **g** Nuclear and cytosolic lysates from A549 and H1299 cells transfected with sh*CIP2A* were separated, and the total PKM2 was determined by western blot. Nuclear lamin B and cytoplasmic tubulin were used as controls. Cyto, cytoplasm; Nuc, nucleus. **h** The cells were transfected with siNC or si*CIP2A*, lysed 48 h later, and the lysates were assayed for pyruvate kinase activity. One-way ANOVA; *P < 0.05, **P < 0.01. **i**, **j** H1299 cells stably expressing *CIP2A* shRNA were treated with vehicle (DMSO) or 30 μM TEPP-46 for 36 h and subsequently subjected to pyruvate kinase activity (**i**) and ECAR (**j**) analyses. Data represents means ± SD of three independent experiments. Two-sided unpaired Student’s *t*-test; *P < 0.05, **P < 0.01, ***P < 0.001; n.s. not significant.

We tested how CIP2A regulates PKM2, and found that knockdown or overexpression of CIP2A had no effect on the total PKM2 protein level (Supplementary Fig. S3a, b). We then tested whether CIP2A influences PKM2 oligomerization. Interestingly, size exclusion chromatography indicated that PKM2 formed tetramer in A549 cells (Fig. 3a, upper panel), whereas silencing of CIP2A by transfection of sh*CIP2A* into the cells markedly reduced PKM2 tetramer (Fig. 3a, upper panel). In H1299 cells, PKM2 tetramer was also detected and was reduced by sh*CIP2A* transfection (Fig. 3a, middle panel) and increased by CIP2A overexpression (Fig. 3b, lower panel). To confirm these observations, Flag-*CIP2A* and HA-*PKM2* were transfected into 293T cells, and the results showed that CIP2A overexpression skewed PKM2 towards high-molecular-weight forms (Fig. 3b). Crosslinking experiments revealed that PKM2 migrated as a single band at ~60 kDa (monomer) in the absence of crosslinking reagent, indicating that SDS treatment resulted in a complete dissociation of potential PKM2 multimers (Fig. 3c). Following crosslinking conditions (0.01% glutaraldehyde at 37 °C for 9 min), tetrameric (240 kDa) PKM2 was increased after CIP2A overexpression in H1299 cells (Fig. 3c). Furthermore, the tetramer was significantly reduced and replaced by dimeric (120 kDa) and monomeric forms when CIP2A was knocked down in H1299 cells (Fig. 3d). To validate whether CIP2A influences the interaction between PKM2 in cells, sh*CIP2A* and HA-*PKM2* were transfected and we found that the amount of endogenous PKM2 that coprecipitated with HA-PKM2 was reduced in sh*CIP2A*-transfected H1299 cells (Fig. 3e). These observations suggested that CIP2A facilitates PKM2 tetramer assembly.

### CIP2A determines PKM2 cellular localization and pyruvate kinase activity

PKM2 normally localizes in the cytoplasm, and also non-canonically localizes in the nucleus and mitochondria^46^. To test the effect of CIP2A on the cellular distribution of PKM2, we analyzed confocal microscopy images by comparing the PKM2 signal intensity overlapping with 4′,6-diamidino-2-phenylindole (DAPI) staining (nuclear region) by ZEN software^47^. A higher-intensity PKM2 signal from the nucleus was visualized in H1299 cells after knockdown of CIP2A (Fig. 3f). Consistent with these findings, western blot analysis of subfractionated cellular compartments indicated that CIP2A deficiency substantially increased nuclear PKM2 in H1299 and A549 cells (Fig. 3g). The mild reduction of PKM2 in the cytosolic fractions upon CIP2A knockdown may be attributed to the fact that the nuclear PKM2 is indeed a small portion of the total PKM2 (Fig. 3g). These results suggested that CIP2A inhibits the nuclear translocation of PKM2.

We assessed whether CIP2A could regulate PKM2 pyruvate kinase activity, and found that silencing of CIP2A by si*CIP2A* decreased PKM2 pyruvate kinase activity by 50%–70% in lysates of CIP2A-depleted H1299 and A549 cells (Fig. 3h). Metabolic flux analysis was further carried out to test the involvement of PKM2 in metabolic reprogramming regulated by CIP2A. The specific PKM2 activator TEPP-46^48^ increased pyruvate kinase activity (Fig. 3i) and reduced ECAR (Fig. 3j), whereas knockdown of CIP2A suppressed pyruvate kinase activity (Fig. 3i) and increased ECAR (Fig. 3j) in the presence or absence of TEPP-46 in H1299 cells. Consistent with the altered glycolysis phenotype, CIP2A deficiency-induced mitochondrial respiration reduction, as measured by OCR, was blocked by TEPP-46 treatment (Supplementary Fig. S3c). Additionally, TEPP-46 also reversed the acidic culture medium induced by CIP2A depletion in A549 cells (Supplementary Fig. S3d). These data suggested that CIP2A was able to regulate PKM2 pyruvate kinase activity.

### PP2A regulatory subunit B56α controls PKM2 activity and reprograms glycolysis

**Fig. 4:**
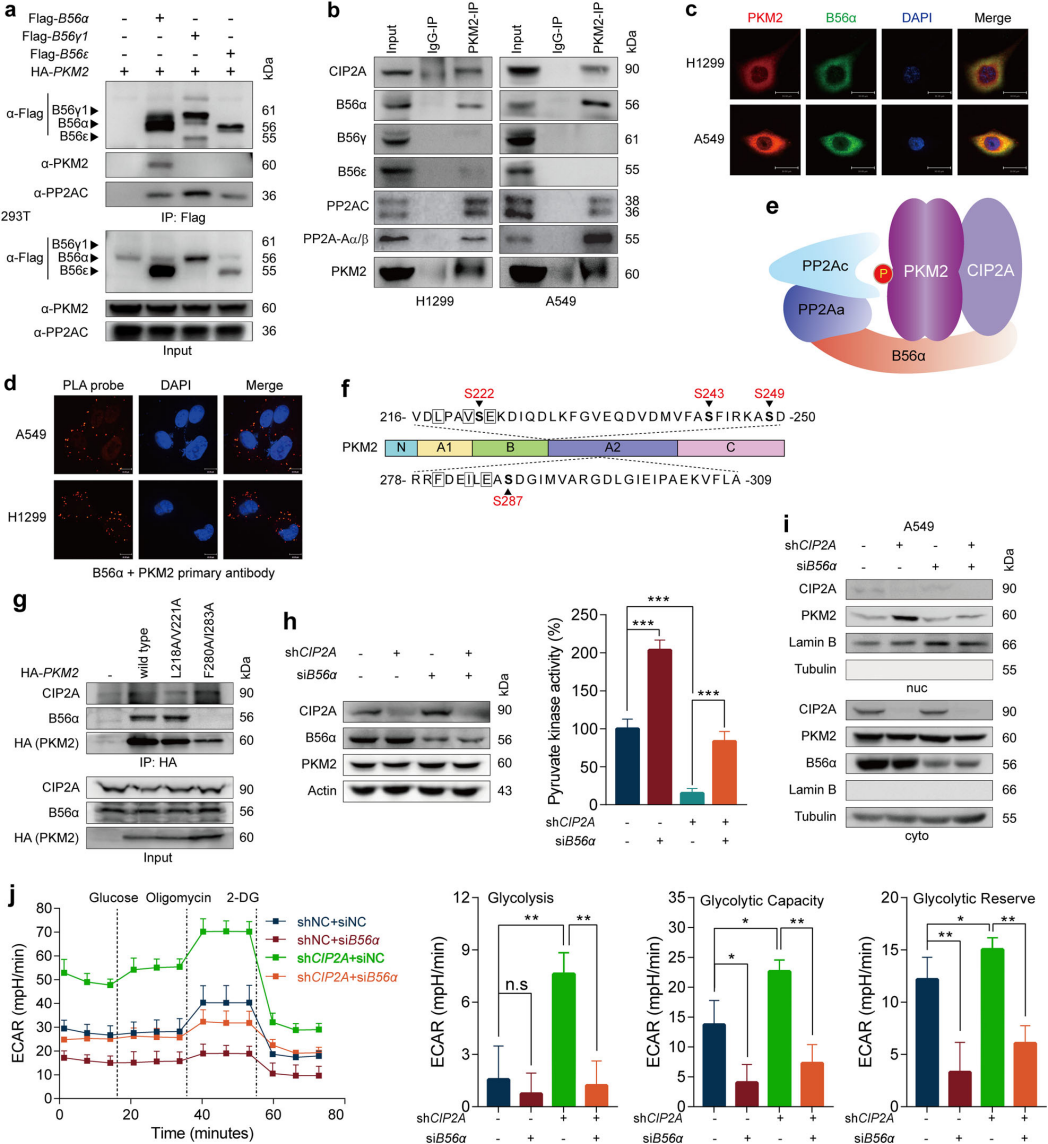
B56α regulates PKM2 activity and metabolism. **a** The indicated constructs were transfected into 293T cells, and the total protein extracts were immunoprecipitated and analyzed by western blot. **b** Co-IP of endogenous PKM2 in A549 and H1299 cells. The co-immunoprecipitated proteins were analyzed by western blotting using the indicated antibodies. **c** IF assays of H1299 and A549 cells using antibodies against PKM2 (red) and B56α (green), and DAPI to stain nuclei (blue). Scale bars, 30 μm. **d** Representative images of Duolink in situ PLA with B56α and PKM2 primary antibodies in A549 cells and H1299 cells. Scale bars, 10 μm. **e** Schematic representation of the ternary complex composed of the PP2A complex, PKM2 and CIP2A. **f** Schematic representation of the protein domains of PKM2 and the location of two potential B56α binding motifs and surrounding serine that might be modified. **g** The indicated HA-*PKM2* mutant plasmids were transfected into H1299 cells, and proteins immunoprecipitated by HA-PKM2 were analyzed by western blotting. **h**–**j** A549 cells stably expressing *CIP2A* shRNA were transfected with siRNA targeting *B56α* or control for 36 h, and then pyruvate kinase activity (**h**), subcellular localization of PKM2 (**i**) and ECAR (**j**) were determined. One-way ANOVA; *P < 0.05, **P < 0.01, ***P < 0.001; n.s. not significant. Cyto cytoplasm, Nuc nucleus.

We found that si*CIP2A* transfection resulted in reduced tetrameric and increased dimeric/monomeric PKM2 (Fig. 3a and Supplementary Fig. 4a). Meanwhile, knockdown of PP2A A subunit by siRNA slightly increased tetrameric and reduced dimeric/monomeric PKM2, and co-transfection of si*PP2A-Aα*/*β* inhibited the effects induced by CIP2A depletion (Supplementary Fig. S4a). The B family of PP2A regulatory subunits is composed of a large array of different members, and only a subset of the PP2A subunit directly interacts with and is inhibited by CIP2A^49^. We tested which B subunit mediates the modulation of PKM2 activity by CIP2A, and found that only B56α (PPP2R5A), but not B56γ (PPP2R5C) or B56ε (PPP2R5E), interacts with ectopic HA-PKM2 (Fig. 4a). Co-IP analysis of endogenous proteins confirmed that B56α interacted with PKM2 (Fig. 4b). IF analysis demonstrated the colocalization of B56α and PKM2 in A549 and H1299 cells (Fig. 4c), and PLA assays confirmed the interaction between B56α and PKM2 (Fig. 4d). PKM2 also interacted with both PP2AC and PP2A-Aα/β in H1299 and A549 cells (Fig. 4b). These results suggested that CIP2A, PKM2 and PP2A might form ternary complex (Fig. 4e) to regulate cell metabolism.

A short linear motif, [LMFI]xx[ILV]xEx (where x is any amino acid), acts as the preferred docking site for the complementary pocket conserved in the B56 subunit, whereas substitution of the two best consensus residues at positions 1 and 4 to alanine would remarkably reduce the binding affinity^50,51^. We therefore analyzed the amino acid sequence of PKM2 and found two possible B56α binding motifs in the A2 domain (Fig. 4f). We constructed two mutated versions of the two potential binding motifs and found that F280A/I283A, but not L218A/V221A, completely disrupted the interaction between B56α and HA-PKM2, which could be the binding site (Fig. 4g).

We tested the effects of B56α on PKM2 pyruvate kinase activity and found that depletion of B56α notably upregulated the pyruvate kinase activity, in the presence or absence of sh*CIP2A* (Fig. 4h). Importantly, B56α deficiency substantially blocked CIP2A depletion-elicited PKM2 nuclear distribution (Fig. 4i). Inhibition of CIP2A repressed PKM2 pyruvate kinase activity (Fig. 4h) and increased ECAR (Fig. 4j) in the presence or absence of si*B56α*, and vice versa, si*B56α* transfection upregulated pyruvate kinase activity and inhibited ECAR in the presence or absence of sh*CIP2A*. In parallel, the increased OXPHOS level (Supplementary Fig. S4b) and the yellow discoloration of the culture medium (Supplementary Fig. S4c) due to CIP2A depletion were also reversed by co-depletion of B56α. These results suggested that CIP2A-PP2A B56α axis is important in regulating pyruvate kinase activity and ECAR.

### PKM2 serine 287 is a novel phosphorylation site

**Fig. 5:**
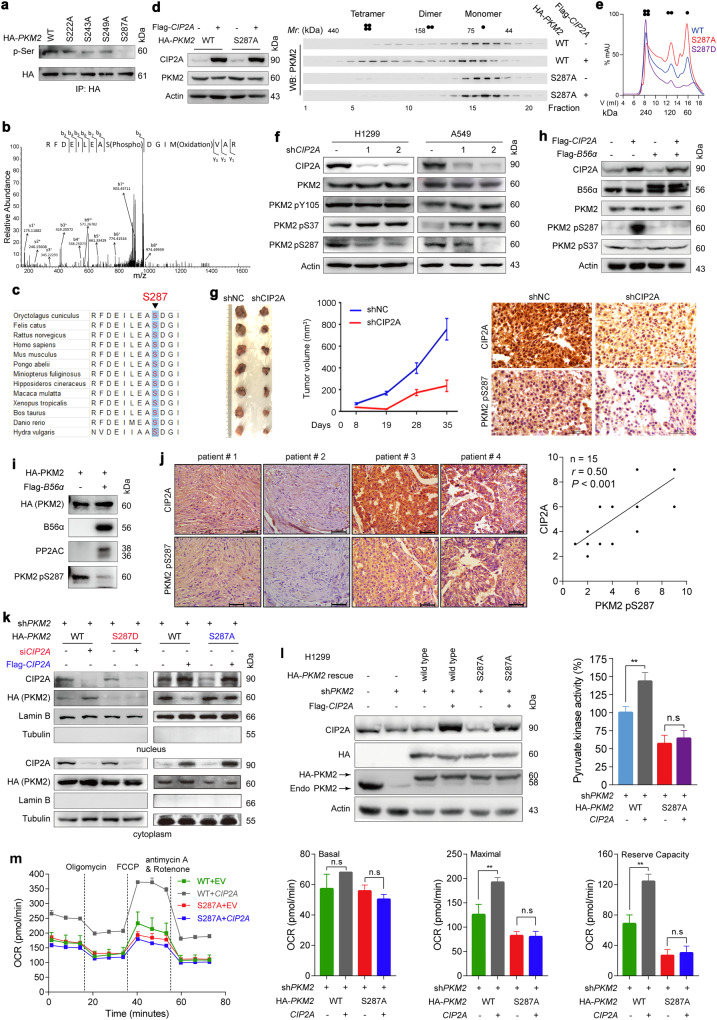
S287 is a critical phosphorylation site of PKM2. **a** S287A decreases PKM2 phosphorylation. The indicated HA-*PKM2* mutant plasmids were transfected into 293T cells, and protein was immunoprecipitated for phosphorylation analysis. **b** MS shows PKM2 S287 phosphorylation. **c** S287 of PKM2 is evolutionarily conserved in the indicated species. The sequences around PKM2 S287 from different species were aligned. **d** CIP2A shifts WT PKM2 but not S287A mutant PKM2 to higher-molecular-weight fractions. The indicated plasmids were transfected into 293T cells, and cell lysates were separated by size exclusion chromatography, followed by western blot analysis. Fraction numbers and relative molecular weights (Mr) are indicated. Ectopic expression of Flag-CIP2A and HA-tagged WT PKM2 or S287A PKM2 mutant in 293T cells was detected by western blot. **e** Size exclusion chromatography of purified recombinant WT, S287A and S287D PKM2 that were expressed in bacteria. The molecular mass standards and theoretical molecular mass of PKM2 tetramer (240 kDa)/dimer (120 kDa) are marked on the lower axis. **f** Western blot analyses of PKM2 phosphorylation at Y105, S37 and S287 in A549 and H1299 cells transfected with sh*CIP2A*. **g** Knockdown of CIP2A inhibits tumor growth and suppresses PKM2 S287 phosphorylation in tumor samples of mice inoculated with sh*CIP2A*-expressing A549 cells. A549 cells transfected with shNC are used as a control. **h** Western blot analysis of PKM2 phosphorylation at S287 and S37 in H1299 cells that were transfected with Flag-*CIP2A* and Flag-*B56α*. **i** Effects of B56α on phosphorylation of PKM2 S287 by an in vitro dephosphorylation assay using HA-tagged PKM2 and FLAG-B56α proteins purified from 293T cells. **j** IHC staining experiments were performed in 15 human lung adenocarcinoma specimens using anti-CIP2A and anti-PKM2 pSer287 antibodies, and the correlation between immunoreactivity scores (IRSs) of CIP2A and PKM2 pS287 was analyzed. Note that some scores overlap, and the dots on the graphs indicate no less than one sample. Scale bars, 50 μm. **k** Cytosolic and nuclear fractions were prepared from H1299 cells transfected with sh*PKM2*, shRNA-resistant HA-*PKM2* (WT or S287D), and si*CIP2A*. Nuclear lamin B and cytoplasmic tubulin were used as controls. **l**, **m** Flag-*CIP2A* and reconstituted shRNA-resistant HA-*PKM2* (WT or S287A mutant) were transfected into sh*PKM2*-treated H1299 cells. Pyruvate kinase activity (**l**) and OCR mitochondrial respiration parameters (**m**) were measured. PKM2 silencing and re-expression efficiency were verified by western blot. Data represent means ± SD of three independent experiments. EV empty vector. Two-sided unpaired Student’s *t-*test; **P < 0.01; n.s. not significant.

We next assessed the potential B56α-mediated posttranslational modifications of PKM2 by generation of Ser-to-Ala substitutions near the two motifs and transfection into 293T cells. The mutant proteins were purified by IP followed by western blotting with an anti-phosphoserine antibody. We found that S287A mutation significantly reduced PKM2 phosphorylation, while the other 3 mutants (S222A, S243A, S249A) did not significantly reduce the overall phosphorylation of PKM2 (Fig. 5a). Additionally, MS analysis also suggested that S287 is the most promising candidate serine phosphorylation site (Fig. 5b), and a genomic analysis showed that S287 is highly conserved among different species during evolution (Fig. 5c), implying that S287 could be a major phosphorylation site under this condition.

To study whether the phosphorylation status at S287 affects CIP2A/PP2A-mediated PKM2 dimer-tetramer conversion, we fractionated 293T cell lysates by gel filtration analysis and found that the wild-type (WT) PKM2 was distributed throughout multiple fractions and that co-expression of CIP2A further switched PKM2 from monomer and dimer to tetramer (Fig. 5d). Notably, S287A PKM2 was mainly detected in a low-molecular-weight fraction, and ectopic expression of CIP2A failed to markedly shift fractions of S287A PKM2 to higher-molecular-weight complexes compared with that seen in WT PKM2 (Fig. 5d). These results indicated a strong effect of phosphorylation at S287 in promoting tetramerization of PKM2. To determine the direct role of S287 phosphorylation in dynamic states of PKM2, we performed the size exclusion chromatography with purified recombinant PKM2 variants^48^, and found that WT PKM2 existed as a mixture of tetramer, dimer and monomer, and peaked in tetramer form (Fig. 5e). While S287D PKM2 mainly existed as tetramer, S287A peaked in monomeric and dimeric forms (Fig. 5e).

### CIP2A modulates PKM2 phosphorylation at S287

To study whether S287 is phosphorylated in vivo, we generated an antibody specifically against phospho-S287 (pS287) and carried out a series of experiments to test its specificity. The dot plot assay showed that the PKM2 pS287 antibody preferentially detected the phosphorylated peptide but not the unmodified peptide and the signal gradually increased with increasing peptide concentration (Supplementary Fig. S5a). Western blotting and immunohistochemistry (IHC) assays indicated that the staining was absent when the antibody was preincubated and neutralized with the phosphorylated peptide compared with the unmodified peptide (Supplementary Fig. S5b, c). In parallel, when PKM2 was knocked down by expression of a short hairpin RNA (shRNA), the band disappeared, as detected by the pS287 antibody (Supplementary Fig. S5d). These results verified the specificity of the antibody.

We showed that CIP2A silencing remarkably downregulated pS287 in H1299 and A549 cells (Fig. 5f). To test this observation in vivo, sh*CIP2A*-transfected A549 cells were injected into right flank of nude mice. We found that compared to cells transfected with shNC, sh*CIP2A*-transfected cells grew more slowly in mice and the tumor volume was smaller (Fig. 5g). The IHC assays showed that the PKM2 pS287 level in tumor samples of mice inoculated with sh*CIP2A*-transfected cells was lower than that in mice injected with shNC-transfected A549 cells (Fig. 5g). We tested the effect of PP2A on PKM2 pS287, and found that coexpression of B56α inhibited CIP2A-induced PKM2 S287 phosphorylation (Fig. 5h), which was confirmed by an in vitro dephosphorylation assay using Flag-B56α and HA-PKM2 proteins purified from 293T cells (Fig. 5i).

We carried out IHC staining analysis of 15 primary human lung adenocarcinoma tissue samples using a NSCLC tissue microarray (Supplementary Table S1) to examine the clinical relevance of PKM2 pS287. We observed that pS287 predominantly localized to the cytoplasm and was hardly detected in the nucleus (Fig. 5j, left panel). Notably, the levels of PKM2 pS287 and CIP2A were correlated with each other (Fig. 5j, right panel).

### S287 phosphorylation is critical for PKM2 activity

To obtain insights into the function of PKM2 pS287, we replaced endogenous *PKM2* with either shRNA-resistant HA-*PKM2*, the phospho-mimetic mutant S287D HA-*PKM2*, or S287A HA-*PKM2* in H1299 cells (Fig. 5k, l). Subcellular fractionation analysis indicated that S287D PKM2 mainly localized in the cytoplasm and CIP2A depletion-induced nuclear translocation was inhibited (Fig. 5k). Compared to WT HA-PKM2-expressing cells, HA-PKM2 S287A cells presented lower pyruvate kinase activity (Fig. 5l), lower OXPHOS (Fig. 5m), and higher ECAR (Supplementary Fig. S5e), and was unable to be modulated by CIP2A ectopic expression (Fig. 5l, m; Supplementary Fig. S5e). Additionally, H1299 cells expressing HA-PKM2 S287A maintained more acidic culture medium regardless of CIP2A expression (Supplementary Fig. S5g). On the contrary, S287D PKM2 exhibited lower ECAR and higher OCR (Supplementary Fig. S5e). These results revealed that PKM2 phosphorylation at S287 is required for CIP2A-induced changes in PKM2 oligomerization, pyruvate kinase activity and glucose flux.

### The phosphorylation of S287 in PKM2 modulates the nuclear localization of the protein

**Fig. 6:**
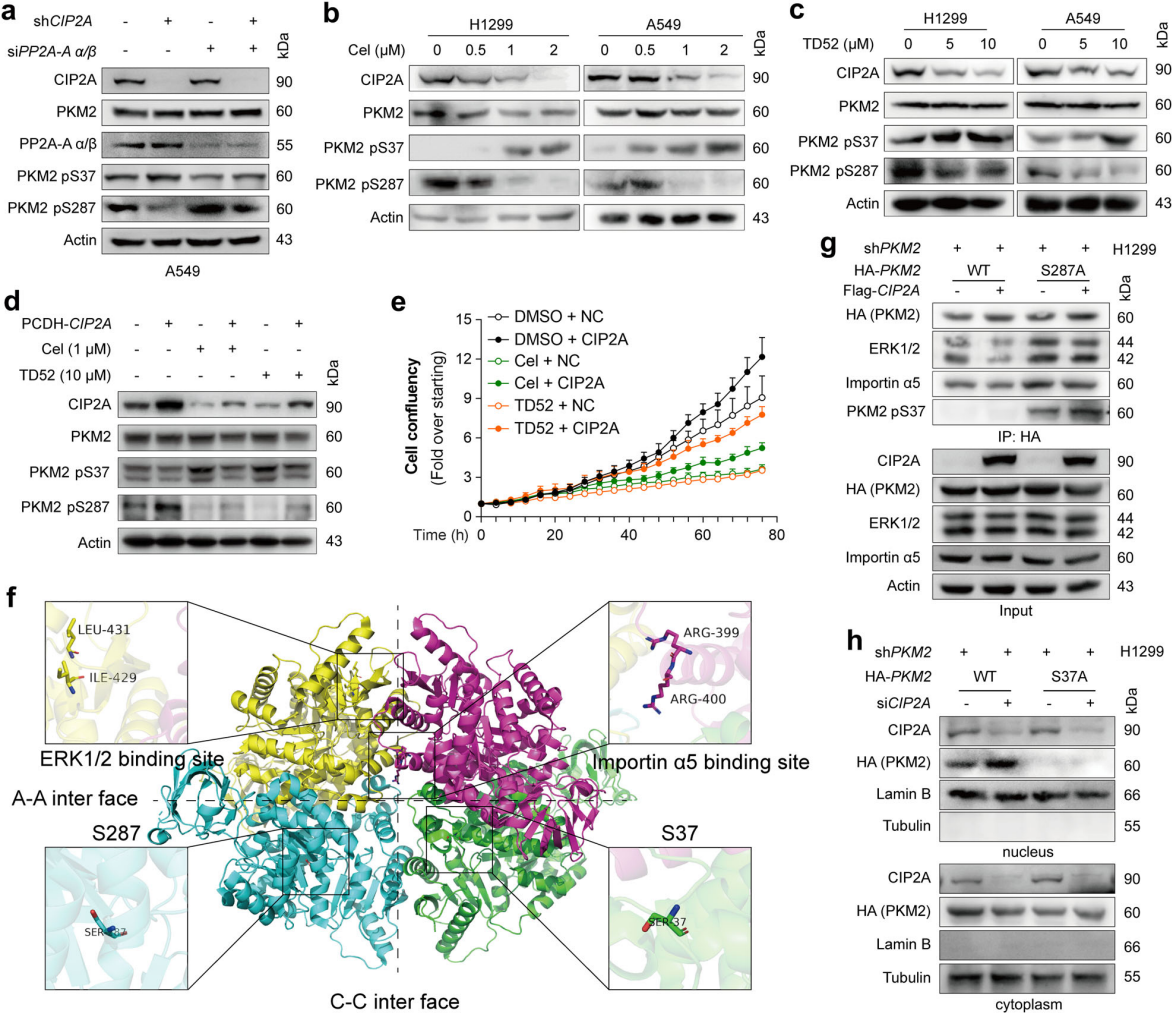
CIP2A induces PKM2 phosphorylation at S287 and nuclear translocation. **a** Knocking down PP2A blocks CIP2A depletion-induced S37 phosphorylation. A549 cells stably expressing *CIP2A* shRNA were transfected with *PP2A-A α/β* siRNA or control, followed by immunoblotting analysis. **b**, **c** H1299 and A549 cells were treated with celastrol (**b**) or TD52 (**c**) for 48 h, lysed, and cell lysates were analyzed by immunoblotting. **d**, **e** H1299 cells were transfected with exogenous *CIP2A*, and 48 h later treated with celastrol/TD52 for additional 48 h. The cells were lysed for western blot using the indicated antibodies (**d**) or cell proliferation was measured by an IncuCyte Live-Cell Analysis System (**e**). **f** Structural analysis of the human PKM2 tetramer (PDB code: 3SRD). The four PKM2 monomers are shown in cartoon mode with different colors, and the interfaces between two dimers are represented by dashed lines. The amino acid residues Ile429/Leu431 (ERK1/2 binding sites), Arg399/400 (importin α5 binding site), Ser287 and Ser37 are indicated by ball and stick models and colored yellow, purple, cyan and green, respectively. Magnified images with labeled residues are also presented. **g** Interaction among PKM2, ERK1/2, importin α5 and p-PKM2(S37). Flag-*CIP2A* and shRNA-resistant HA-tagged *PKM2* (WT or S287A mutant) were transfected into PKM2-depleted H1299 cells, followed by IP and western blotting analysis with the indicated antibodies. **h** Cytosolic and nuclear fractions were prepared from H1299-sh*PKM2* cells transfected with shRNA-resistant HA-*PKM2* (WT or S37A mutant) and si*CIP2A*. Nuclear lamin B and cytoplasmic tubulin were used as controls.

We further used the other two available phospho-specific antibodies against PKM2 to determine whether it is influenced by CIP2A. In H1299 and A549 cells, we found that CIP2A depletion had no effect on the phosphorylation level of PKM2 Y105 but significantly increased S37 phosphorylation and downregulated S287 phosphorylation (Fig. 5f). We tested whether these observations were associated with PP2A, and found that ectopic expression of CIP2A reduced phospho-S37 (pS37) and increased pS287, which was reversed by coexpression of B56α (Fig. 5h); conversely, depletion of the PP2A A subunit blocked CIP2A silencing-induced pS37 upregulation and pS287 downregulation (Fig. 6a).

We used two small compounds, celastrol that was shown to be able to trigger proteasomal degradation of CIP2A^27^, and TD52 that is a compound derived from EGFR inhibitor erlotinib and could reduce CIP2A expression without impacting EGFR activity^52,53^, to test the effects of CIP2A on PKM2 pS37 and pS287. We found that in H1299 and A549 cells, treatment with 0.5–2 μM celastrol for 48 h downregulated CIP2A in a dose-dependent manner (Fig. 6b). While the expression level of the total PKM2 protein was not perturbed, pS37 PKM2 was markedly upregulated and pS287 PKM2 was substantially downregulated by celastrol (Fig. 6b). Similarly, TD52 treatment downregulated CIP2A and pS287 PKM2 and upregulated pS37 PKM2 in the cells (Fig. 6c). These effects could be partially inhibited by ectopic expression of CIP2A (Fig. 6d). Moreover, while celastrol and TD52 inhibited the proliferation of H1299 cells, exogenous CIP2A partly inhibited this effect (Fig. 6e). The exogenous CIP2A was unable to completely antagonize the effects of celastrol and TD52, probably attributed to the fact that this transcript was not a degradation-resistant CIP2A.

Previous studies indicated that the phosphorylation of PKM2 at S37 was ERK1/2 dependent and could promote PKM2 binding to importin α5 and translocation to the nucleus^40^. Interestingly, crystal structure analysis of human PKM2 (PDB code: 3SRD) revealed that the ERK1/2 binding sites (Ile429/Leu431) and the pivotal residues Arg399/400 of the nuclear localization sequence (NLS), which binds to importin α5, are buried in the prominent C–C interface (Fig. 6f). However, CIP2A-induced tetramerization may obstruct PKM2 accessibility to ERK1/2 and importin α5, and the dimeric PKM2 is likely exposed to recruit ERK1/2 and importin α5, which promotes S37 phosphorylation and PKM2 nuclear translocation. We found that the ectopic expression of CIP2A caused a decrease in the binding of HA-PKM2 to endogenous ERK1/2 and importin α5, whereas the HA-PKM2 S287A mutant, which existed in a dimer state, reinforced this binding and manifested a high level of pS37 and were unaffected by transfection with Flag-CIP2A (Fig. 6g). Consistent with the previous observation^40^, S37A PKM2 failed to translocate into the nucleus (Fig. 6h); surprisingly, S37A mutant also abrogated PKM2 nuclear translocation induced by CIP2A knockdown (Fig. 6h). These results suggested that both the phosphorylated S37 and non-phosphorylated S287 are crucial for nuclear translocation of PKM2.

### CIP2A redirects PKM2 to mitochondria to upregulate Bcl2 expression

**Fig. 7:**
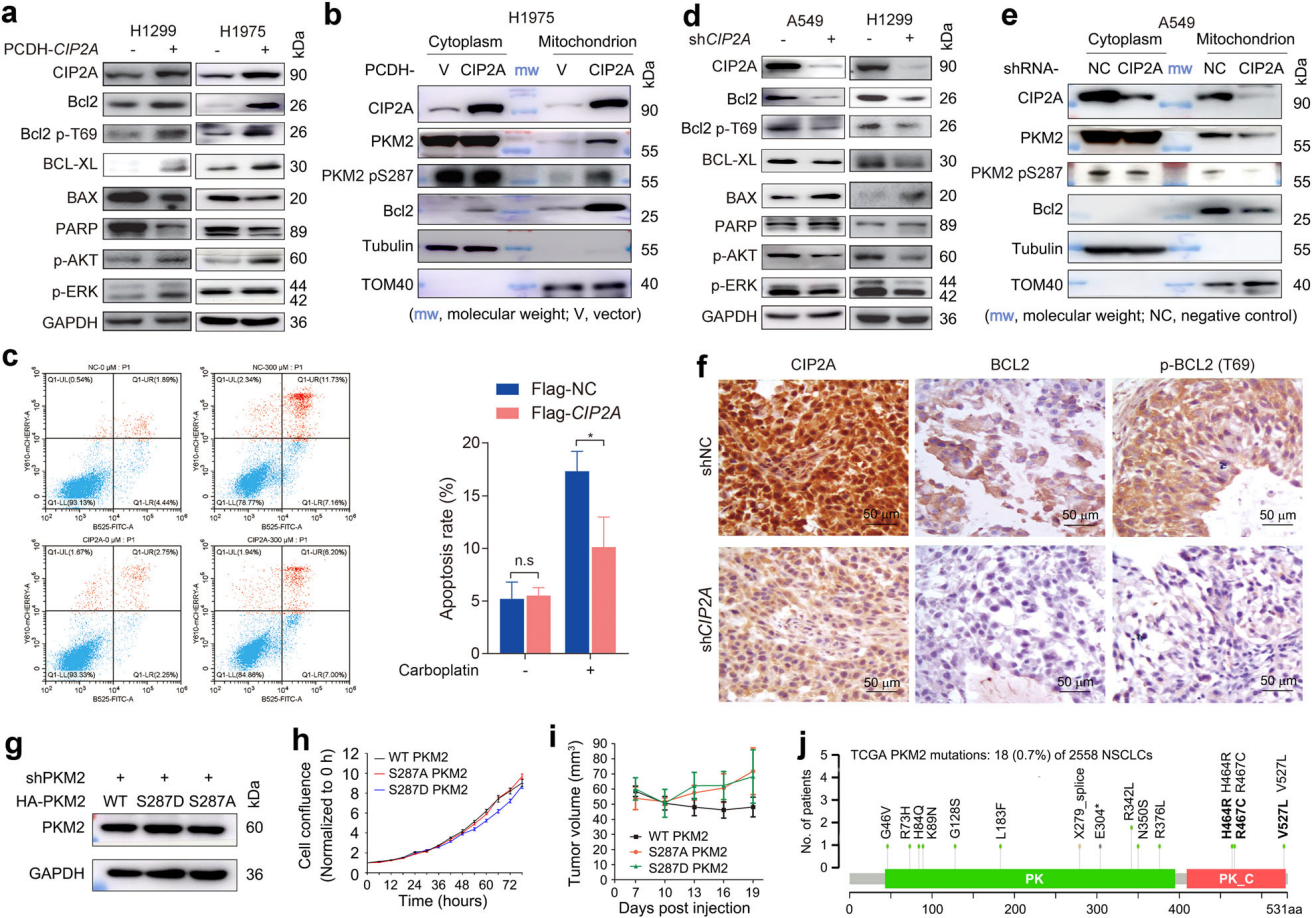
CIP2A upregulates Bcl2 in NSCLC cells. **a** H1299 and H1975 cells were transfected with pCDH-*CIP2A*, lysed 48 h later, and cell lysates were subjected to western blot using the indicated antibodies. **b** H1975 cells were transfected with pCDH-*CIP2A*, lysed 48 h later, and fractions isolated from different compartments were subjected to western blotting using the indicated antibodies. **c** pCDH-*CIP2A*-expressing H1299 cells were treated with carboplatin and cell apoptosis was evaluated with Annexin V-FITC/propidium iodide method. **d** Western blot analyses of the indicated proteins using lysates from A549 cells that were transfected with sh*CIP2A*. **e** A549 cells were transfected with sh*CIP2A*, lysed 48 h later, and fractions isolated from different compartments were subjected to western blotting. **f** A549 cells that were transfected with sh*CIP2A* were inoculated into the right flank of nude mice (5 ×10^6^ cells/mouse), which were euthanized 37 days after cell injection. Tumors were isolated and tumor sections were obtained for IHC assays using the indicated antibodies. **g** H1299 cells stably expressing sh*PKM2* were transfected with WT, S287D PKM2, or S287A PKM2, and lysed for western blot analysis. **h** Cell proliferation was analyzed by an IncuCyte Live-Cell Analysis System. **i** The cells were inoculated into right flank of the mice, and tumor volume was measured. *n* = 9 for each group. **j** Mutations of PKM2 in TCGA NSCLCs. PK, pyruvate kinase, barrel domain (43–395); PK_C: pyruvate kinase, α/β domain (409–529).

PKM2 regulates oxidative stress-induced apoptosis by stabilizing apoptosis antagonist Bcl2, via interaction with and phosphorylation of Bcl2 at T69^54^. We tested the effect of CIP2A on Bcl2 expression, and found that in H1299 and H1975 cells transfected with CIP2A, the total Bcl2 as well as Bcl2 p-T69 was upregulated (Fig. 7a). Bcl-Xl and p-Akt were also upregulated, whereas Bax was downregulated (Fig. 7a). Proteins in different cellular compartments were isolated, and CIP2A was detected in mitochondrial compartment (Fig. 7b). Exogenous expression of CIP2A upregulated PKM2 in mitochondrial compartment (Fig. 7b). Interestingly, mitochondrial Bcl2 was also upregulated in cells transfected with pCDH-*CIP2A* (Fig. 7b). When the cells were treated with chemotherapy agent carboplatin (0.3 mM) for 48 h, ~10% of the *CIP2A*-overexpressing and Bcl2-upregulated H1299 cells (Fig. 7a) underwent apoptosis, much less than that in carboplatin-treated H1299 cells (Fig. 7c). On the contrary, sh*CIP2A* treatment led to downregulation of Bcl2, Bcl2 p-T69, Bcl-Xl, and p-Akt, and upregulation of Bax (Fig. 7d). Knockdown of CIP2A indued downregulation of mitochondrial PKM2, Bcl2, and Bcl2 p-T69 (Fig. 7e). When sh*CIP2A*-transfected A549 cells were inoculated into nude mice, downregulation of Bcl2 and Bcl2 p-T69 in tumor tissues were detected (Fig. 7f). We investigated the role of S287 phosphorylation in tumor-promoting activity of PKM2 using PKM2 S287D and S287A mutants, and found that in H1299 cells in which endogenous PKM2 was silenced by sh*PKM2*, re-expression of WT, S287D PKM2 and S287A PKM2 conferred approximately equal proliferation speed to the cells (Fig. 7g, h). When the cells were inoculated into nude mice (*n* = 9 for each group), those tumors bearing S287A PKM2 and S287D PKM2 grew slightly but not significantly faster than WT PKM2-harboring tumors (Fig. 7i). In The Cancer Genome Atlas (TCGA) datasets, no mutation was found in S287 of PKM2 (Fig. 7j).

### PKM2 S287 phosphorylation is associated with clinical outcome of the patients

**Fig. 8:**
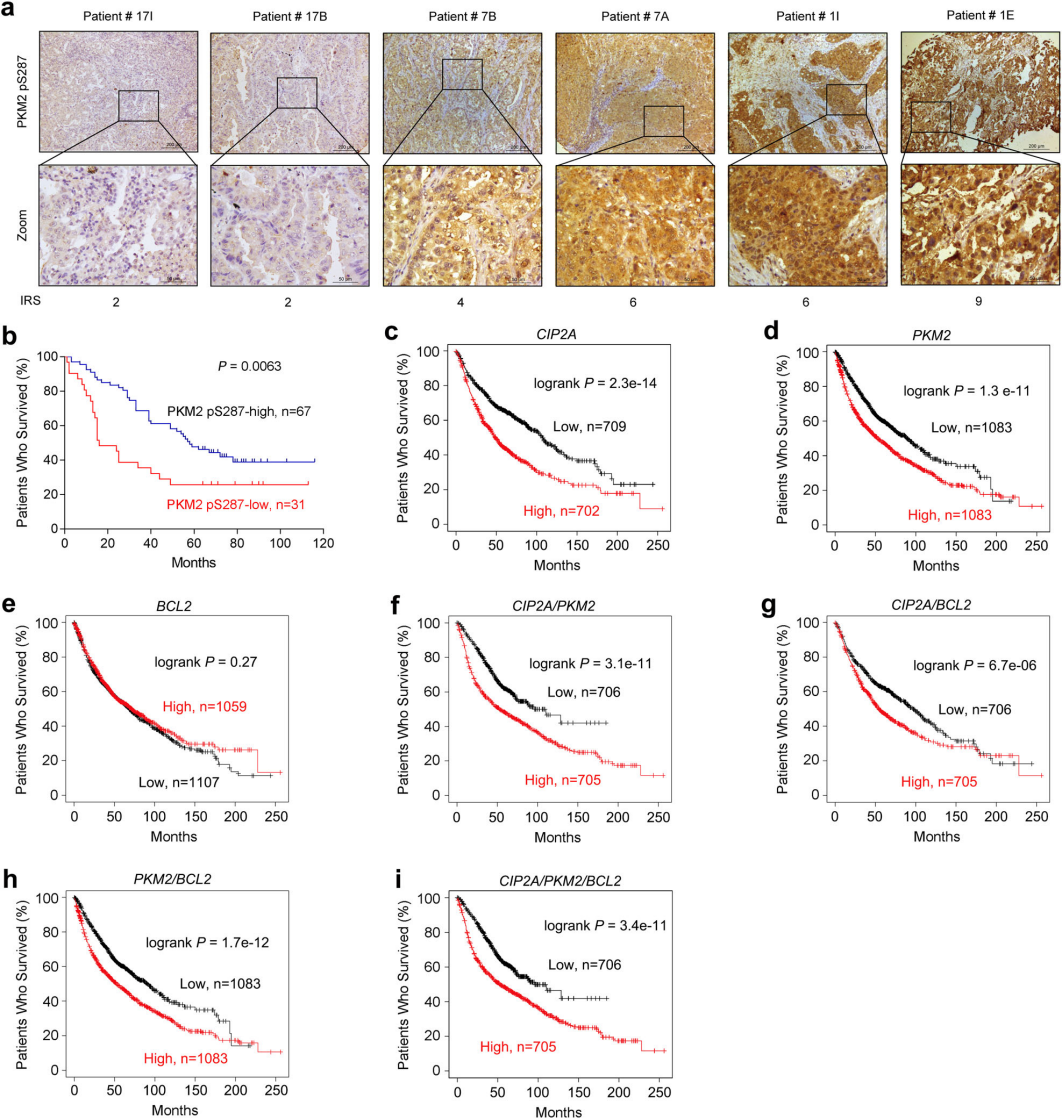
PKM2 expression and clinical outcome of the patients. **a** Representative images of IHC assays of tumor tissues harvested from 98 patients with treatment-naïve lung adenocarcinoma. **b** Overall survival of lung adenocarcinoma patients with high and low PKM2 pS287 levels. The *P* values were calculated using the log-rank test. **c**–**i** Overall survival of NSCLCs with high or low expression levels of *CIP2A*, *PKM2*, *BCL2*, and their combinations. Data were obtained from the Online Survival Analysis Software (http://kmplot.com/analysis/index.php?p=service&cancer=lung).

We tested the PKM2 pS287 levels with IHC assay in tumor samples isolated from 98 patients with treatment-naïve lung adenocarcinoma (Table 1), and the immunoreactivity score (IRS) was calculated. The IRS of these patients was 4.12 ± 2.29 (mean ± SD), and those with IRS < 4.12 were considered PKM2 pS287 low, while those with IRS ≥ 4.12 were considered PKM2 pS287 high. We found that 31 (31.6%) of the patients had lower PKM2 pS287 IRS and worse prognosis than those with higher PKM2 pS287 IRS (67/98, 68.4%; Table 1 and Fig. 8a, b). In addition, by using the Online Survival Analysis Software^55^ (http://kmplot.com/analysis/index.php?p=service&cancer=lung), we found that patients with high expression levels of *CIP2A* (Fig. 8c) and *PKM2* (Fig. 8d) but not *BCL2* (Fig. 8e) had poor prognosis. Patients with high expression levels of *CIP2A*/*PKM2*, *CIP2A/BCL2*, *PKM2/BCL2*, and *CIP2A/PKM2/BCL2* also had poor prognosis (Fig. 8f–i).

**Table 1:** Baseline characteristics of the 98 patients with lung adenocarcinoma.

	Total	Low PKM2 pS287	High PKM2 pS287	*P* value^a^
*n*	98	31	67	
Age				
≤ 60	50	15	35	0.72
> 60	48	16	32	
Sex				
Male	59	19	40	0.88
Female	39	12	27	
Histology				
Adenocarcinoma	93	30	63	0.76
Adenosquamous carcinoma	4	1	3	
Mucoepidermoid carcinoma	1	0	1	
Greatest tumor diameter				
< 4	44	14	30	0.97
≥ 4	54	17	37	
Lymph node involvement				
Negative	46	11	35	0.12
Positive	51	19	32	
Unknown	1	1	0	
Histologic grade				
≤ II	61	19	42	0.89
> II	37	12	25	
Stage				
I–II	55	13	42	0.07
III–IV	42	17	25	
Unknown	1	1	0	

^a^Tested by Fisher’s exact test.

### Combinatory modifications of CIP2A and PKM2 inhibit cell proliferation in vitro and in vivo

**Fig. 9:**
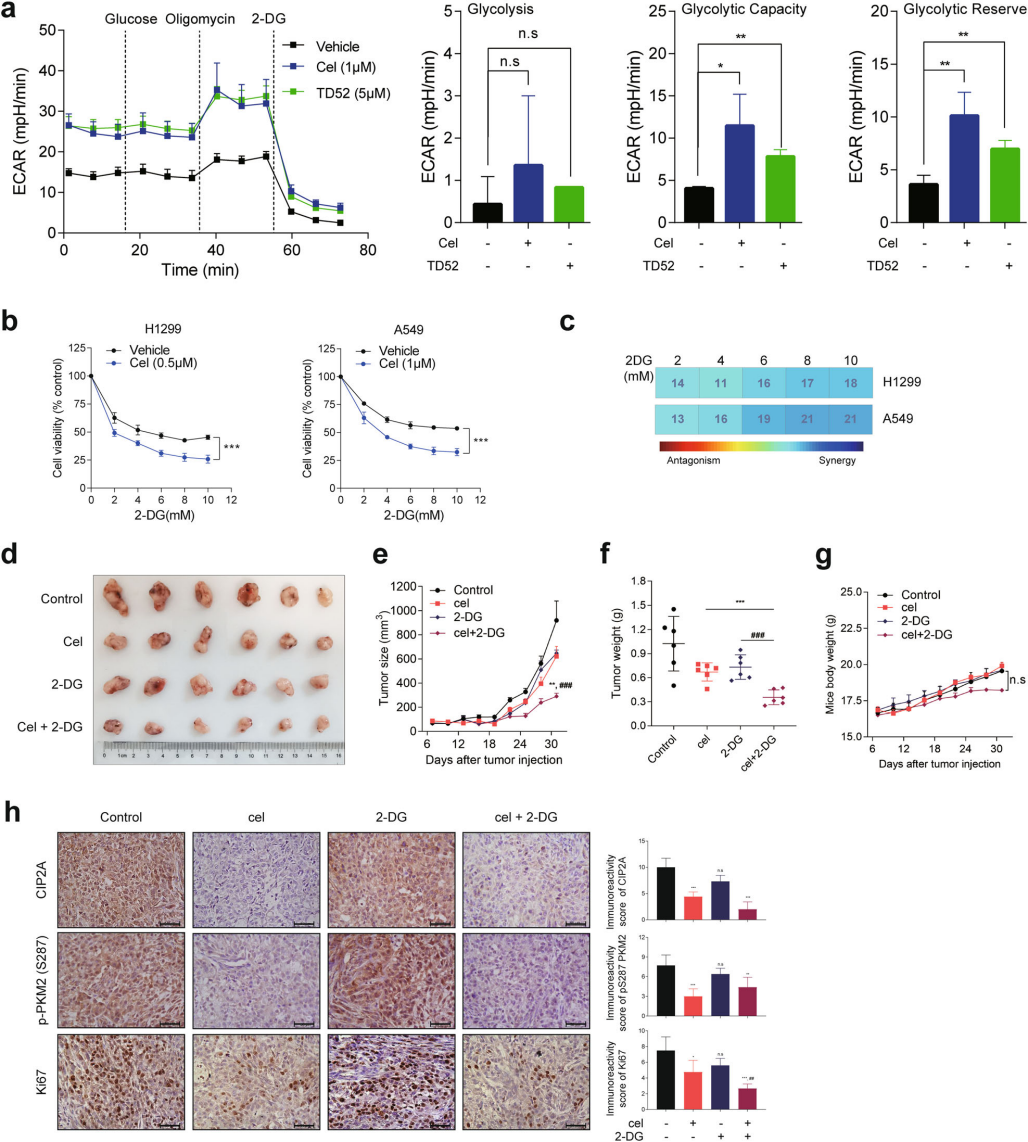
Effects of combinatory modulations of PKM2 and CIP2A on NSCLC cells in vitro and in vivo. **a** ECAR and glycolytic parameters were determined in A549 cells treated with celastrol (1 μM) or TD52 (5 μM). Two-sided unpaired Student’s *t*-test; *P < 0.05, **P < 0.01; n.s., not significant. **b**, **c** Cell viability of H1299 and A549 cells treated with the indicated compounds (**b**), and the combinatory effects were analyzed by Bliss synergism analysis (**c**). Two-way ANOVA; ***P < 0.001. **d**–**g** Nude mice bearing A549 cells were treated with celastrol (2 mg/kg, orally daily) and/or 2-DG (500 mg/kg/day, intraperitoneal injection), and tumor volume was measured every 3 days. Ex vivo images of resected tumors (**d**), growth curves of the tumor volume (**e**), tumor weight (**f**) and body weight (**g**) are shown, respectively. Two-tailed Student’s *t*-test; **P < 0.01, ***P < 0.001 indicate combination group vs celastrol group; ^###^*P* < 0.001 indicates combination group vs 2-DG group. n.s. not significant. **h** Representative IHC staining images for CIP2A, phospho-PKM2 at S287 and Ki67 expression in resected xenograft tumors (left panel). The IRSs were quantified (right panel). Scale bars, 50 μm. Two-sided unpaired Student’s *t*-test; *P < 0.05, **P < 0.01, ***P < 0.001 (indicated group vs control group); ^##^*P* < 0.01 (combination group vs 2-DG group); n.s. not significant.

CIP2A promotes cancer cell growth and in vivo tumor formation^14^, and its inhibition by siRNA (Fig. 5g) or small compounds suppressed the proliferation of lung cancer cells in vitro and in vivo^27,43,44^. We tested the effects of these compounds on glycolysis and OXPHOS, and found that treatment of A549 cells with CIP2A degrons celastrol and TD52 resulted in increased level of glycolysis (measured by ECAR) and decreased level of OXPHOS (measured by OCR) (Fig. 9a and Supplementary Fig. 6a), suggesting the role of CIP2A in PKM2 dimer-tetramer switching and potential benefits from combinatory use of CIP2A degrons and glycolysis inhibitor. We tested these possibilities by treating H1299 and A549 cells with celastrol in combination with glycolysis inhibitor 2-DG, and found that 2-DG enhanced the inhibitory effects of celastrol in these cells (Fig. 9b). 2-DG also enhanced the inhibitory effects of TD52 in both H1299 and A549 cells (Supplementary Fig. S6b). A Bliss synergism analysis was conducted, and the results showed that combined use of celastrol and 2-DG (Fig. 9c) or TD52 and 2-DG (Supplementary Fig. S6c) exerted synergistic effects in inhibition of NSCLC proliferation. In addition, crystal violet staining experiment revealed that dual treatment showed much stronger effects of clonogenic growth suppression than each single agent treatment in A549 and H1299 cells (Supplementary Fig. S6d).

We next examined the in vivo antitumor efficiency of this combination treatment in a xenograft model. We observed that monotherapy with either celastrol or 2-DG at low dosage had a modest effect on tumor growth (Fig. 9d–f) and did not affect body weight of the mice (Fig. 9g). Monotherapy using these agents inhibited tumor cell proliferation, as indicated by Ki67 staining, compared to the controls (Fig. 9h). Importantly, celastrol/2-DG combination exhibited markedly enhanced inhibition of tumor growth (Fig. 9d–f) and suppression of cell proliferation as reflected by the expression level of Ki67 (Fig. 9h), compared to each monotherapy. We found that in tumor tissues isolated from mice treated with celastrol alone or celastrol/2-DG, CIP2A and PKM2 pS287 were substantially decreased (Fig. 9h). These results indicated that glycolysis inhibition was able to synergize with CIP2A-targeted therapy in suppressing NSCLC cell proliferation in vivo.

**Fig. 10:**
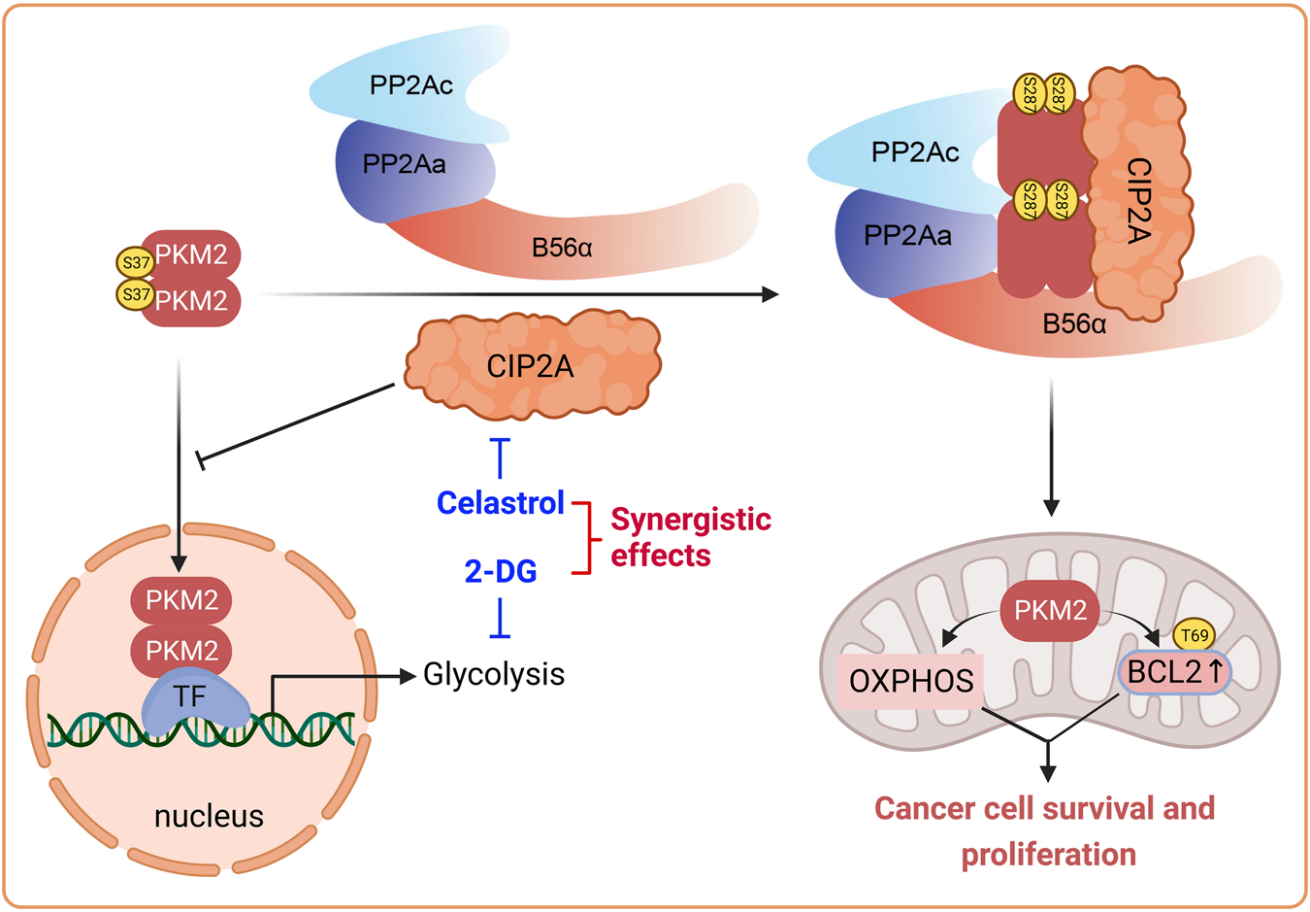
Schematic representation of CIP2A in inducing PKM2 conformational changes and biological functions. CIP2A binds to PKM2 and alters PKM2 phosphorylation by modulating PP2A B56α, leading to PKM2 S287 phosphorylation and tetrameric formation. PKM2 is therefore redirected to mitochondrion, where it enhances OXPHOS, and phosphorylates Bcl2 at T69 and stabilizes Bcl2, resulting in enhanced cancer cell survival and proliferation.

## Discussion

Lung cancer is the leading cause of cancer-related deaths worldwide, which accounts for 18% (~1.8 million) of all cancer deaths in 2020^56^. In recent decades, targeted therapies based on oncogenic driver mutations or fusion proteins (i.e., EGFR and anaplastic lymphoma kinase (ALK)) have increased the overall survival and changed the treatment paradigm of NSCLC (comprising lung adenocarcinoma, lung squamous cell carcinoma, and lung large cell carcinoma)^57^. Immune checkpoint blockades (ICBs), such as antibodies against programmed cell death 1 (PD-1) and programmed cell death-ligand 1 (PD-L1), have improved outcomes of patients without EGFR or ALK mutations, but only ~20% of patients achieve a durable response^58–60^. Currently, the 5-year survival rate for NSCLC with all stages combined is only 18.6%^56,61^. Therefore, novel therapeutic approaches or combination therapies are still urgently needed.

Human NSCLC tumors have enhanced glucose oxidation^4,62^, but the mechanisms remain to be addressed. We found that in NSCLC cells CIP2A knockdown significantly increased the glycolysis rate and lactate production and inhibited OXPHOS, whereas CIP2A overexpression suppressed glycolysis and increased OXPHOS. CIP2A simultaneously impinged PKM2 metabolic and non-metabolic functions through inhibition of the PP2A regulatory B56α subunit. CIP2A-facilitated PKM2 S287 phosphorylation fostered PKM2 tetramer formation and hindered its interaction with ERK1/2 and importin α5 and downstream S37 phosphorylation for PKM2 nuclear translocation. These results indicated that the oncoprotein CIP2A induces metabolic reprogramming by promotion of OXPHOS and inhibition of aerobic glycolysis in NSCLC cells. Since it is overexpressed in a majority of NSCLCs^44^, CIP2A might be a crucial oncoprotein that promotes OXPHOS in NSCLC cells.

The activity of the metabolic enzyme PKM2 is associated with its multimerization status and subcellular location, which could be regulated by posttranslational modifications, including phosphorylation. Nuclear PKM2 functions as a coactivator of β-catenin to induce the expression of c-Myc, which upregulates glucose transporter 1 (GLUT1), lactate dehydrogenase A (LDHA), and PKM2 itself, leading to enhanced aerobic glycolysis^40^. PKM2 interacts with mitofusin 2 (MFN2)^63^ to promote mitochondrial fusion and OXPHOS, and attenuate glycolysis. Moreover, PKM2 is methylated by a coactivator-associated arginine methyltransferase-1 (CAMR1) and translocates to mitochondrial-associated endoplasmic reticulum membrane (MAM), where it downregulates inositol 1,4,5-trisphosphate receptors (InsP3Rs) and reduces mitochondrial membrane potential and Ca^2+^ uptake, leading to enhancement of OXPHOS^36^. Tyr105 and Ser37 are two well-explored modification sites that are stimulated by the tyrosine kinase fibroblast growth factor receptor (FGFR) and EGFR-ERK1/2, respectively^33,40^. It has been reported that the dual-specificity phosphatase Cdc25A can dephosphorylate PKM2 pS37 only in the nucleus^64^. In this study, we first demonstrated that CIP2A induced PKM2 tetramer formation and enhanced its pyruvate kinase activity through inhibition of PP2A, and the PP2A regulatory subunit B56α controls dephosphorylation of PKM2 pS287 in the cytoplasm. Since EGFR is a binding partner of CIP2A (https://thebiogrid.org/121687/summary/homo-sapiens/kiaa1524.html), the role of EGFR-ERK1/2 in phosphorylation of S287 of PKM2 warrants further investigation. Although B56 family members harbor a highly conserved substrate-interacting surface-exposed pocket^50,51^, B56α but not B56γ or B56ε could bind to PKM2. In addition, PKM2 is a new substrate of PP2A B56α, though it can bind many substrate proteins^50,51^. Few molecules have been shown to be able to promote PKM2 tetramer, and CIP2A represents an oncoprotein that controls PKM2 dimer-tetramer switching.

Enhanced glycolysis is common among NSCLC tumors^4^, while recent studies show that NSCLC tumors also have enhanced OXPHOS^4–7^. We found that PKM2 S287A mutation that inhibits pyruvate kinase activity, blocks PKM2 nuclear localization and enhances glycolysis, did not further promote tumor growth, possibly due to its impaired OXPHOS, and the balance between glycolysis and OXPHOS could be important for tumor growth. Similarly, PKM2 S287D mutation that exhibited impaired glycolysis activity, did not enhance its cancer-promoting activity, also suggesting the importance of the balance between glycolysis and OXPHOS. In addition, PKM2 is a conservative protein, whose mutation rate in TCGA datasets is only 18 (0.7%) in 2558 NSCLCs, suggesting the importance of WT PKM2 protein in lung carcinogenesis.

CIP2A is overexpressed in most human cancers, including lung, breast, colon, gastric, prostate cancers and neck and head carcinomas,^14,15,44,65,66^ and is inversely correlated with disease outcome in NSCLC^43,67^, gastric cancer^66^, ovarian cancer^68^ and chronic myeloid leukemia (CML)^69^. CIP2A promotes tumorigenesis and is associated with drug resistance. It has been identified as a major determinant of EGFR tyrosine kinase inhibitor erlotinib-induced apoptosis in NSCLC cells without EGFR mutation^70^. Additionally, CIP2A-PP2A-Akt signaling was constitutively activated in erlotinib-resistant cells and could mediate drug resistance to erlotinib in EGFR-mutated NSCLC cells^71^. Indeed, inhibition of CIP2A by siRNA/shRNA or small compounds suppress cell proliferation in vitro and tumor growth in vivo^14,27,43,44^. However, we found that CIP2A inhibition by shRNA and small compounds inhibited OXPHOS and prompted NSCLC cells to undergo glycolysis through dephosphorylation of PKM2 pS287, indicating that cancer cells might undergo metabolic reprograming in response to therapeutic stress. These results also suggested that combinatory use of glycolysis inhibitor (e.g., 2-DG) may enhance the anti-lung cancer effects of CIP2A inhibitor. We tested this possibility and found that celastrol in combination with 2-DG did result in markedly enhanced antitumor effects of celastrol, providing a rational combinatory therapeutic strategy for this deadly disease.

In summary, we identified CIP2A as a novel binding partner and modulator of PKM2. CIP2A facilitates PKM2 transformation from dimer to tetramer and redirects it to mitochondrion, where PKM2 upregulates Bcl2 by phosphorylating Bcl2 at T69 and stabilizing Bcl2. Mechanistically, phosphorylation of PKM2 at the S287 site is critical for CIP2A to regulate PKM2 functions and to suppress glycolysis in NSCLC cells (Fig. 10). The combination of a glycolysis inhibitor with the CIP2A-targeting compound celastrol exhibits a synergistic antitumor effect and could serve as a novel and promising strategy for treating patients with NSCLC.

## Materials and methods

### Cell culture and transfection

Embryonic kidney HEK293T and NSCLC cell lines A549, H1299, and H1975 (American Type Culture Collection, Manassas, VA, USA), human SCLC cell lines, H446, H69, H82, and H526 were cultured in DMEM or RPMI 1640 medium supplemented with 10% fetal bovine serum (HyClone, Logan, UT, USA) at 37 °C in a 5% CO_2_/95% air incubator. Transient transfection of plasmids or siRNAs was conducted using the Lipofectamine 3000 kit (Invitrogen) according to the vendor’s instructions. All siRNAs were synthesized by GenePharma (Shanghai, China) and the sequences are shown in Supplementary Table S2.

### Plasmids and mutagenesis

PCR-amplified human *CIP2A*, *B56α*, *B56γ1* and *B56ε* were cloned into the pcDNA3.1-Flag vector. PCR-amplified human *PKM2* cDNA and *PKM2* truncated mutants were cloned into the pcs2-HA vector. GST-*CIP2A* was cloned into the pGEX-4T-1 vector. *PKM2* mutants, including L218A/V221A, F280A/I283A, S222A, S243A, S249A, S287A, S287D and S37A, were generated using a QuikChange site-directed mutagenesis kit (Stratagene, La Jolla, CA, USA). shRNA-resistant *PKM2* was generated by introducing nonsense mutations in shRNA-targeting sites comprising C1170T, C1173T, T1174C and G1176T. Core plasmids used to generate *CIP2A* and shRNA-resistant HA-*PKM2* (WT, S287A and S287D) stable expression cell lines were constructed in pCDH-CMV-GFP and pLVX-IRES-Neo vectors, respectively.

### Virus packaging and stable cell line generation

The shRNAs were cloned into the pLKO.1 vector. The target sequences are listed in Supplementary Table S2. Lentiviral vectors for gene knockdown by shRNA or open reading frame gene expression were transfected into 293T cells together with packaging plasmid (psPAX2) and envelope plasmid (pMD2.G) using the Lipo3000 kit (Invitrogen) according to the manufacturer’s instructions. After 36 h and 60 h, the viruses were collected, filtered, and used to infect target cells in the presence of 8 μg/mL polybrene for 12 h. The infected cells were selected by puromycin or G418 and evaluated by western blotting.

### Seahorse metabolic assays

A549 or H1299 cells were seeded into an XF96 cell culture plate (Seahorse Bioscience) at a density of 1 × 10^4^ cells per well and incubated with complete medium overnight. ECAR and OCR were detected by a Seahorse XFe-96 Analyser (Agilent, Cheadle, Greater Manchester, UK). To monitor changes in ECAR, 10 mM glucose, 1.0 μM oligomycin and 50 mM 2-DG were used. Changes in OCR were assessed by sequential injection of final concentrations of 1.0 μM oligomycin, 0.5 μM FCCP and 0.5 μM rotenone and antimycin A. Corresponding metabolic parameters were further calculated by WAVE Software (Seahorse Bioscience, Agilent) and normalized based on the cell number of each well.

### MS

The CIP2A complex was obtained by IP with anti-CIP2A antibody from a 15-cm culture dish of A549 and H1299 cells. Cells were lysed in IP buffer (40 mM Tris-HCl, pH 7.4, 150 mM NaCl, 1.5 mM MgCl_2_, 0.5% nonidet P-40, 2 mM EDTA, and 1 mM PMSF, 1 mM proteinase inhibitor cocktail) and immunoprecipitated with Protein A/G PLUS-Agarose overnight at 4 °C. The beads were washed five times with IP buffer. The eluted proteins were resolved by SDS-PAGE, silver stained, and the gel bands of proteins were excised for in-gel digestion, and the proteins were identified using MS. Briefly, proteins were treated with 25 mM DTT and alkylated with 55 mM iodoacetamide to reduce disulfide. Sequencing grade modified trypsin in 50 mM ammonium bicarbonate was used for in-gel digestion at 37 °C overnight. The peptides were extracted twice with 1% trifluoroacetic acid in 50% acetonitrile aqueous solution for 30 min. To reduce the volume, the peptide extracts were centrifuged in a SpeedVac. For LC-MS/MS analysis, peptides were separated by 60 min gradient elution at a flow rate of 0.3 μL/min with a Thermo-Dionex Ultimate 3000 HPLC system, which was directly interfaced with a Thermo LTQ-Orbitrap Velos pro mass spectrometer.

PKM2 posttranscriptional modification sites were analyzed by LC-MS/MS. Briefly, H1299 cells were cotransfected with Flag-CIP2A and HA-PKM2 for 48 h, and cell lysates immunoprecipitated by HA magnetic beads (Cell Signaling Technology) were subjected to 10% SDS-PAGE and proteins were visualized by Coomassie blue staining. The stained bands at a molecular weight of 58 kDa were excised and revealed by LC-MS/MS at Tsinghua University.

### IP, GST pull-down and western blot assays

Cell lysates used for the IP assay were extracted in IP lysis buffer at 4 °C for 30 min. Supernatants were clarified by centrifugation at top speed (16,000× *g*) for 10 min at 4 °C. The supernatants were incubated with the corresponding antibodies (2 μg; Supplementary Table S3) as indicated and 30 μL of Protein A/G PLUS-Agarose (Santa Cruz Biotechnology) at 4 °C overnight. The beads were washed five times with IP lysis buffer. The beads were boiled with 2× SDS-PAGE loading buffer for 6 min. The input lysates and immunoprecipitate were separated by 5%–15% SDS-PAGE and analyzed by western blotting.

Recombinant GST-CIP2A proteins (1–560 aa) were produced in *Escherichia coli* BL21 (DE3) cells and purified with Glutathione Sepharose 4B (GE healthcare). GST or GST-CIP2A proteins (20 μg) were incubated with Glutathione Sepharose 4B for 4 h at 4 °C, followed by incubation with His-PKM2 (Abcam) at 4 °C overnight. Thereafter, the samples were extensively washed five times with 1 mL lysis buffer. Finally, the agarose beads were boiled with SDS-PAGE loading buffer for 5 min and analyzed by western blotting. Nuclear and cytosolic fractions were separated using a Minute^TM^ Cytoplasmic and Nuclear Extraction Kit for Cells (SC-003, Invent Biotechnologies) according to the manufacturer’s protocol. Detailed information of the antibodies used in this study are listed in Supplementary Table S3, and a PKM2 phosphorylation (S287)-specific antibody was generated by immunizing rabbits with S287-phosphopeptide (ILEAS(p)DGI) at ABclonal, Inc.

### IF staining and confocal microscopy

Cells were seeded on cover slides, fixed with 4% formaldehyde, and then permeabilized with 0.3% Triton X-100/PBS at room temperature. Then, the cells were blocked in 5% bovine serum albumin/PBS for 30 min at room temperature and then incubated with primary antibody overnight at 4 °C, followed by incubation with FITC/PE-labeled secondary antibodies for 1.5 h at room temperature. After incubation with DAPI to stain the nuclei, the cells were visualized with a confocal laser scanning microscope (Nikon, N-STORM Super-Resolution). Subcellular fractionation was analyzed with the line profile tool in the LSM 780 META ZEN 2011 software package (Carl Zeiss).

### Size exclusion chromatography

The size exclusion chromatography (gel filtration) column (Superdex 200 Increase 10/300 GL, GE Healthcare) was washed with distilled water and equilibrated with PBS. Cells were lysed in lysis buffer (50 mM Tris, pH 7.5, 150 mM NaCl, 0.3% nonidet P-40, 1 mM PMSF, 1 mM proteinase inhibitor cocktail (Roche)), and 5–7 mg/mL total protein (500 μL) was loaded into the column and eluted with elution buffer phosphate buffered saline (50 mM sodium phosphate, 0.15 M NaCl, pH 7.2). The flow rate was 0.5 mL/min. Every 300 μL fraction was collected, and 20 μL of each fraction was analyzed by western blot. In these experiments, both exogenous PKM2 (542 aa) and endogenous PKM2 (531 aa) were included. The molecular weight of these two types of PKM2 is approximately 59.4 kDa and 57.8 kDa, respectively. Therefore, there will be one or two bands of PKM2 in western blot assays, depending on the electrophoresis time: one band could be detected when the electrophoresis time was relatively short (1 h) and two bands could be detected when the electrophoresis time was relatively long (1.5 h or longer).

### Protein crosslinking assay

A total of 1 × 10^6^ cells were lysed with sodium phosphate buffer (pH 7.3) containing 0.5% Triton X-100, 1 mM PMSF and 1 mM proteinase inhibitor cocktail for 30 min at 4 °C. Crude cell lysates were clarified by centrifugation at top speed (16,000× *g*) for 15 min at 4 °C. For crosslinking reactions, the supernatants ( > 5 mg/mL) were treated with 0.01% glutaraldehyde for 9 min at 37 °C. The reactions were terminated by adding 1 M Tris buffer to a final concentration of 50 mM Tris-HCl (pH 8.0). Samples were then separated by 5%–15% SDS-PAGE and analyzed by western blotting.

### Measurement of pyruvate kinase activity

For the in vitro pyruvate kinase activity assay, PKM2 (5 nM) was co-incubated with various concentrations of GST-CIP2A (0–600 nM). The kinase activity was measured with a colorimetric-based pyruvate kinase activity assay kit (Biovision) according to the manufacturer’s protocol. Whole-cell pyruvate kinase activity was measured with the same kit.

### Cell proliferation and viability

Cells expressing different HA-PKM2 mutants were seeded into 12-well plates, and the cell confluence area was monitored every 4 h over a 3-day period using an IncuCyte Live-Cell Analysis System (Essen Bioscience). To determine cell viability, cells transfected with or without CIP2A siRNA were plated into 96-well plates, and cytotoxic activity was assessed after the indicated drug treatment for 48 h using a Cell Counting Kit-8 (CCK-8; Vazyme) assay. The effects of the indicated compounds (Supplementary Table S3) on the cells were detected by CCK-8 assay, and the potential synergistic effect of the two compounds was calculated by the Bliss model using Combenefit software^72^. For colony forming activity, the cells were plated into 12-well plates and treated with TEPP-46 and/or 2-DG for 48 h, and fixed with 4% paraformaldehyde and stained with 0.1% crystal violet. To evaluate the effects of CIP2A overexpression on cell apoptosis, H1299 cells were transiently transfected with empty vector or Flag-CIP2A, and 24 h later the cells were treated with 0.3 mM carboplatin (APExBIO, Houston, TX, USA) for another 24 h. The cells were stained with Annexin V/FITC-propidium iodide Apoptosis Detection kit (APExBIO, Houston, TX, USA) and analyzed by flow cytometry (BD Biosciences, San Jose, CA, USA).

### In vitro dephosphorylation assay

Flag-*B56α* and HA-*PKM2* constructs were transfected into 293T cells using Lipofectamine 3000 (Invitrogen) according to the manufacturer’s instructions. After being transfected for 36 h, cells were collected and suspended in phosphatase assay buffer (containing 20 mM imidazole-HCl, 2 mM EDTA, 2 mM EGTA, pH 7.0) supplemented with protease inhibitors (phosphatase inhibitors should also be added to the phosphatase assay buffer which was used to extract PKM2 substrate to preserve phosphorylation). Thereafter, the cells were sonicated for 10 s, centrifuged at 2000× *g* for 5 min and the supernatants were incubated with anti-Flag beads (Sigma) or anti-HA beads (Cell Signaling Technology) at 4 °C with rotation overnight. Following 4–5 washes, HA-PKM2 substrate and FLAG-B56α phosphatase were eluted in pNPP Ser/Thr buffer (Sigma) and co-incubated at 30 °C for 30 min. Finally, the phosphatase and substrate mixture were boiled with SDS-PAGE loading buffer for 5 min and analyzed by western blotting.

### PLA

PLA was performed according to the Duolink in situ Fluorescence protocol (Sigma). Briefly, cells were grown on glass coverslips, fixed with 4% paraformaldehyde and permeabilized using 0.1% Triton X-100 in PBS for 20 min at room temperature. Cells were blocked for 1 h at 37 °C with Duolink Blocking Solution and then the blocking solution was replaced with the primary antibodies at 4 °C overnight. The subsequent step was incubating cells with the Duolink in situ PLA Probe Anti-Rabbit PLUS and Anti-Mouse MINUS PLA probes (Sigma) for 1 h at 37 °C. Then, cells were incubated with ligation solution for 30 min at 37 °C and amplification solution for 100 min at 37 °C. Finally, coverslips were mounted onto slides with Duolink in situ Mounting Medium with DAPI (Sigma).

### Xenograft studies

The animal studies were approved by and conducted according to the Ethics Committee of Cancer Hospital, Chinese Academy of Medical Sciences. Five-week-old female nude BALB/c mice were purchased from Vital River Laboratory Animal Technology Co., Ltd. (Beijing, China), kept in a specific pathogen-free environment and used for the animal assays. After 7 days of acclimatization, 5 × 10^6^ A549 cells were subcutaneously inoculated into the dorsal flank. When reaching ~100 mm^3^, the tumor-bearing mice were randomly divided into six groups (6 mice per group) for treatment with the control, celastrol (2 mg/kg orally daily), TEPP-46 (100 mg/kg orally daily), or 2-DG (500 mg/kg/day, intraperitoneal injection), and combination treatment of celastrol with TEPP-46 or 2-DG. Celastrol and TEPP-46 were dissolved in 10% DMSO and 90% CMC-Na (Sigma), and 2-DG was dissolved in normal saline. Tumor volumes were measured every 3 days by electronic calliper and calculated following the formula: volume = 1/2 length × width^2^. Mouse body weights were monitored regularly throughout the course of the animal experiments. Thirty-five days later, the mice were sacrificed, and the tumor tissues were dissected, photographed, weighed and fixed in 4% formaldehyde for further IHC studies.

### Patient samples and IHC assay

The study was approved by the research ethics committee of the Cancer Hospital, Chinese Academy of Medical Sciences and was conducted in accordance with the Declaration of Helsinki. All lung cancer samples were collected with informed consent. The diagnosis of lung cancer was confirmed by at least two pathologists. Human lung cancer tissue arrays were purchased from Shanghai Outdo Biotech (Cat# HLug-Ade030PG-01 and HLugA180Su08), and the samples were obtained from patients (Table 1 and Supplementary Table S1) with written informed consent. IHC staining of resected mouse tumor tissues and human lung cancer tissue arrays was carried out according to the manufacturer’s protocol (Zhongshan Golden Bridge, Beijing, China). The quantification of IHC was calculated as IRS (0–12) = SP (0–1: 0%–25%; 2: 26%–50%; 3: 51%–75% and 4: > 75%) × SI (0: no signal; 1: weak; 2: moderate and 3: strong), where IRS is the immunoreactivity score, SP is the proportion of stained positive cells and SI is the staining intensity. Detailed information about the antibodies used in IHC are listed in Supplementary Table S3.

### Statistical analysis

All experiments were performed at least three times, and the results are presented as the means ± SD. Differences between data groups were evaluated for significance using Student’s *t*-test of unpaired data or one-way ANOVA. *P* < 0.05 (two-sided) was considered statistically significant (*P < 0.05, **P < 0.01 and ***P < 0.001; n.s., not statistically significant).

### Supplementary information

Supplementary information
