# Supplementary material for: CIP2A induces PKM2 tetramer formation and oxidative phosphorylation in non-small cell lung cancer
Source: Cell Discov. 2024 Feb 6;10:13. doi: 10.1038/s41421-023-00633-0 (PMC10847417; doi:10.1038/s41421-023-00633-0)
Supplement: Supplementary file 1 — Supplementary information [file 41421_2023_633_MOESM1_ESM.pdf]

Supplementary Materials for

CIP2A induces PKM2 tetramer formation and oxidative phosphorylation in non-small cell lung cancer

Li-Jun Liang[1,2]*, Fu-Ying Yang[1]*, Di Wang[1,3]*, Yan-Fei Zhang[4]*, Hong Yu[1,5], Zheng Wang[1], Bei-Bei Sun[1], Yu-Tao Liu[1], Gui-Zhen Wang[1#], Guang-Biao Zhou[1#]

This file includes:

Supplementary Tables S1 to S3

Supplementary Figure S1 to S6 and legends

Table S1. Demographics and characteristics of the 15 lung adenocarcinoma patients.

Sample-ID	Gender	Age	Tissue sampling	Pathological classification	Histologic grade	Lympy node involvement	Stage
NRsLug0312A0007	Male	50	Surgical resection	LUAD	II	Negative	II
CRsLug0412A0055	Female	81	Surgical resection	LUAD	II	Positive	I A3
CRsLug0504A0096	Female	71	Surgical resection	LUAD	II	Positive	II ~ III
CRsLug0609A0191	Female	57	Surgical resection	LUAD	II	Positive	II ~ III
CRsLug0612A0219	Male	63	Surgical resection	LUAD	II	Positive	II ~ III
CRsLug0702A0260	Female	56	Surgical resection	LUAD	II	Positive	II ~ III
CRsLug0702A0262	Male	63	Surgical resection	LUAD	II	Positive	II ~ III
CRsLug0702A0264	Female	30	Surgical resection	LUAD	II	Positive	II ~ III
CRsLug0707A0380	Male	-	Surgical resection	LUAD	II	Positive	II ~ III
CRsLug0709A0392	Female	57	Surgical resection	LUAD	II	Positive	II ~ III
CRsLug0804A0507	Female	61	Surgical resection	LUAD	II	Positive	II ~ III
CRsLug0412A0044	Male	60	Surgical resection	LUAD	II ~III	Positive	II ~ III
CRsLug0612A0230	Female	52	Surgical resection	LUAD	II ~III	Positive	II ~ III
NRsLug0312A0002	Female	37	Surgical resection	LUAD	III	Negative	I B
CRsLug0905A0693	Female	50	Surgical resection	LUAD	III	Positive	II ~ III

Table S2. siRNAs and shRNAs used in this study.

**Target**	**Sequence 1 (5' →3')**
si*CIP2A*-1	CUGUGGUUGUGUUUGCACUTT
si*CIP2A*-2	ACCAUUGAUAUCCUUAGAATT
si*B56α*	GCTAACATCTTCCGTACACTT
si*PP2A-Aα*	GCAUCAAUGUGCUGUCAUATT
si*PP2A-Aβ*	CGACUCAACAGUAUUAAGATT
sh*CIP2A-1*	GACAGAAACTCACACGACTAT
sh*CIP2A-2*	GCTAGTAGACAGAGAACATAA
sh*PKM2*	AGGCAGAGGCUGCCAUCUA

Table S3. Antibodies and small compounds used in this study.

Reagents	Source	Identifier	Application	Dilution rate
**Antibodies again proteins**				
PKM2	CST	#4053	WB/IP	1:1000/1:50
PKM1	CST	#7067	WB	1:1000
PP2AC	CST	#2259	WB	1:1000
p-PKM2 Y105	CST	#3827	WB	1:1000
ERK1/2	CST	#9102	WB	1:1000
STAT3	CST	#9139	WB	1:1000
p-STAT3	CST	#9145	WB	1:2000
mouse anti-rabbit IgG (light chain specific)	CST	#93702	WB	1:3000
HA-Tag magnetic beads	CST	#11846	IP	1:20
Ki67	CST	#44092	IHC	1:250
CIP2A	Abcam	#ab99518	WB	1:2000
Myc	Abcam	#ab32072	WB	1:1000
phosphoserine	Abcam	#ab9332	WB	1:100
CIP2A	Abcam	#ab99518	IHC	1:100
B56γ	Santa Cruz	#sc-374380	WB	1:100
B56ε	Santa Cruz	#sc-376176	WB	1:500
PP2A-Aα/β	Santa Cruz	#sc-13600	WB	1:250
lamin B	Santa Cruz	#sc-6216	WB	1:500
p-PKM2 S37	Signalway	#11456	WB	1:750
importin α5	Signalway	#25191	WB	1:1000
B56α	Proteintech	#12675-2-AP	WB	1:750
actin	Proteintech	#66009-1-Ig	WB	1:10000
rabbit anti-Goat IgG	Proteintech	#SA00001-4	WB	1:5000
tubulin	HuaBio	#M1501-1	WB	1:5000
HA tag	MBL	#561	WB	1:3000
Flag tag	Sigma	#f1804	WB	1:1000
FLAG-probe agarose beads	Sigma-Aldrich	#F1804	IP	1:20
p-PKM2 S287	Our lab (developed by ABclonal, Inc, Wuhan, China)		WB/IHC	1:750/1:150

Bcl2	Abcam	#ab182858	WB	1:1000
Bcl2 pT69	ThermoFisher	PA5-118539	WB	1:1000
**Compounds**				
Compound 3k	APExBIO, Houston, TX, USA	#B8217		
2-deoxyglucose	APExBIO, Houston, TX, USA	#B1027		
TEPP-46	MCE (New Jersey, USA)	#HY-18657		
celastrol	MCE (New Jersey, USA)	#S1290		
TD-52	MCE (New Jersey, USA)	#HY-135699		

Supplementary figures and legends

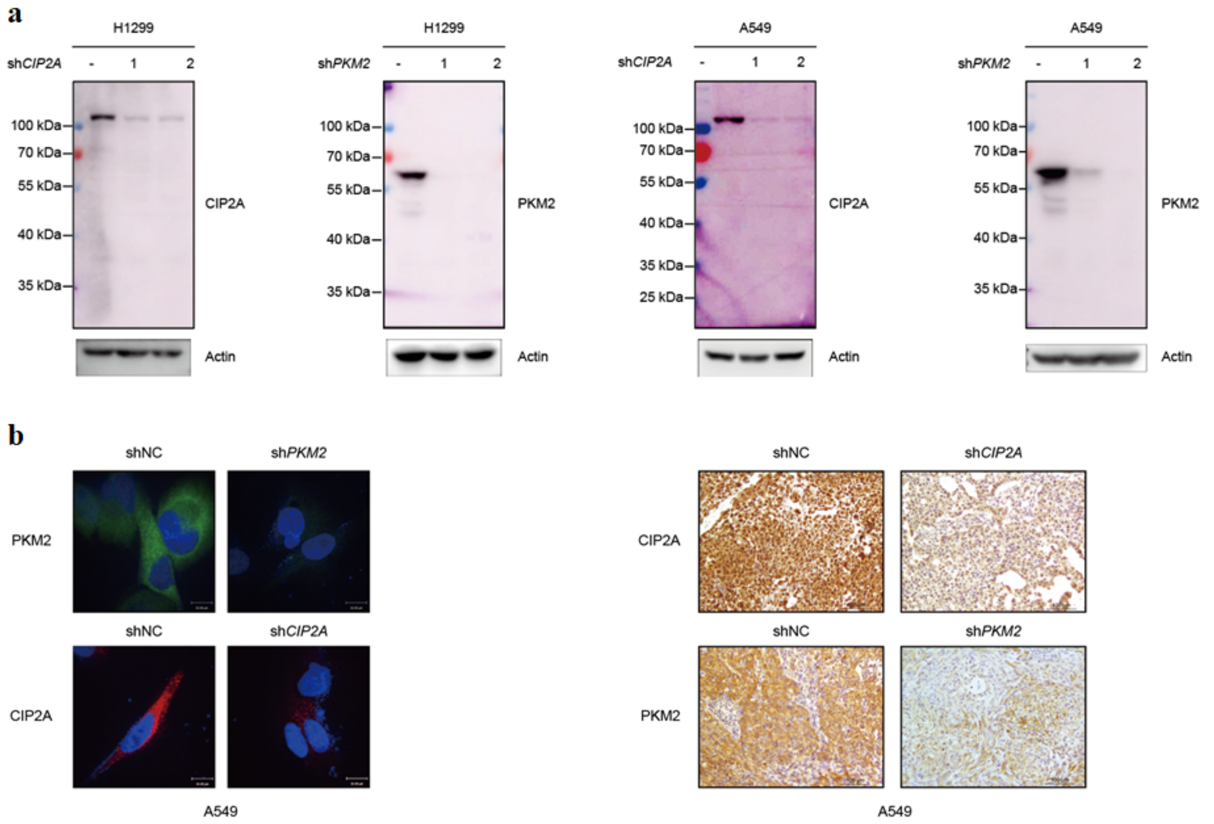

Supplementary Fig. S1. The signals of anti-CIP2A and anti-PKM2 antibodies in Western blot (a), immunofluorescence (b, left) and immunohistochemistry (b, right) assays.

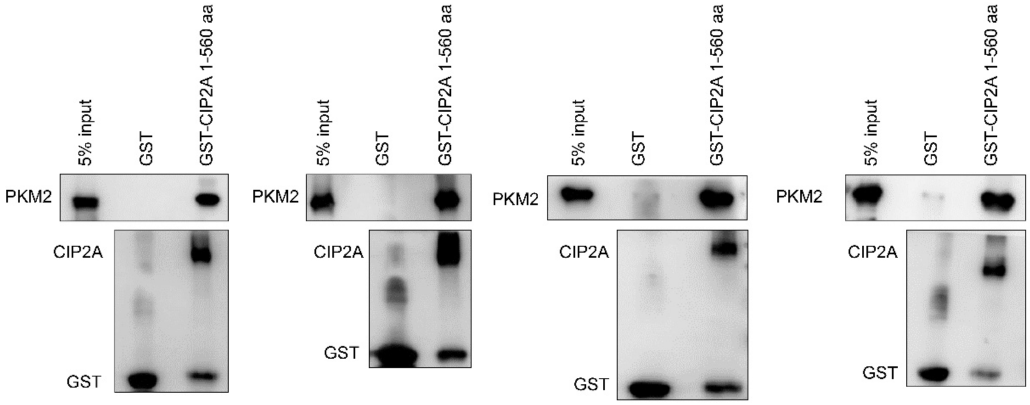

Supplementary Fig. S2. The original data for Fig. 2c of the manuscript. GST pull-down and Western blot assays using indicated antibodies.

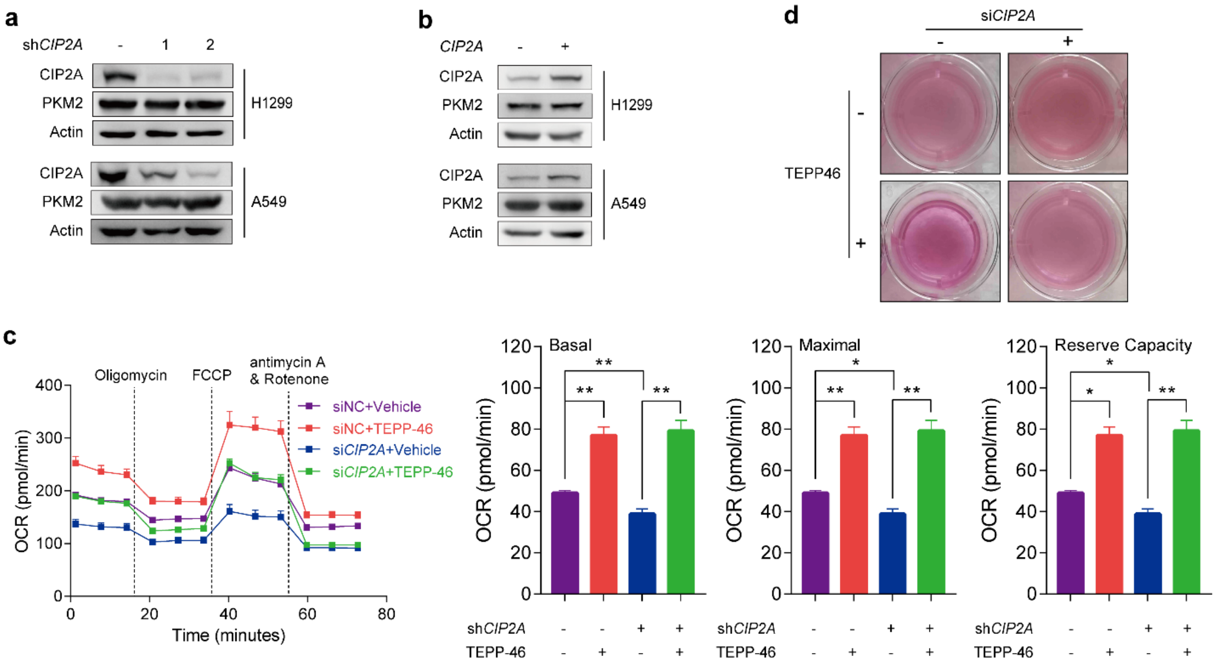

Supplementary Fig. S3. Effects of CIP2A on PKM2 pyruvate kinase activity. (**a, b**) Western blot analysis of PKM2 and CIP2A in H1299 and A549 cells with stable CIP2A knockdown (a) and overexpression (b). (**c**) H1299 cells stably expressing *CIP2A* shRNA were treated with vehicle (DMSO) or 30 μM TEPP-46 for 36 hours and subsequently subjected to OCR analysis. Data represents the average of three independent experiments (mean ± SD). * $P < 0.05$, ** $P < 0.01$. (**d**) Representative cell culture media images of A549 cells transfected with si*CIP2A* with or without concomitant TEPP-46 treatment.

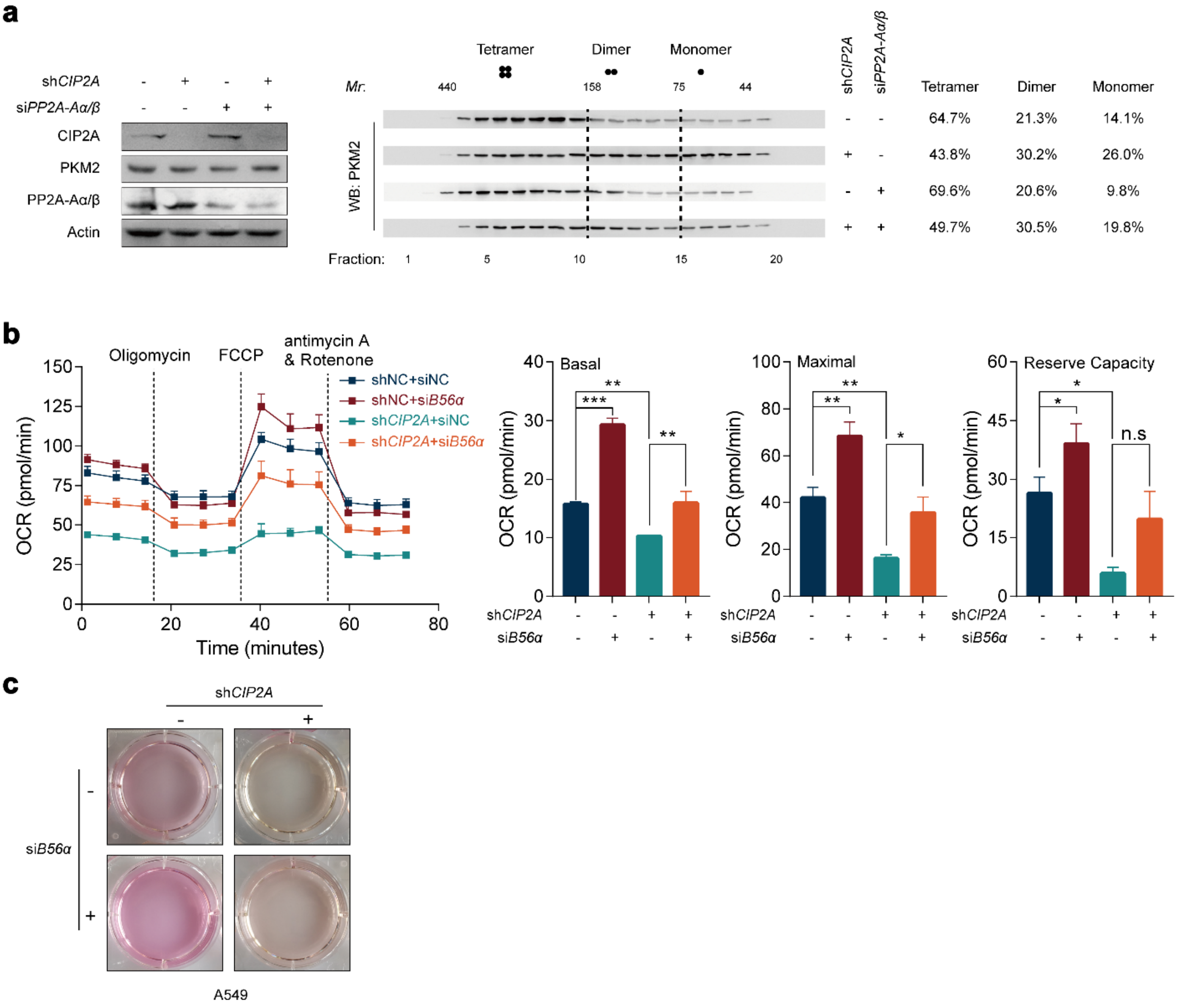

Supplementary Fig. S4. B56α modulates PKM2 activity and metabolic reprogramming in NSCLC cells. (**a**) CIP2A-induced PKM2 tetramerization is dependent on PP2A. A549 cells stably expressing *CIP2A* shRNA were transfected with siRNA targeting PP2A-Aα/β or control for 36 hours, and then the cellular extracts were fractioned by gel filtration, followed by western blot analysis (right panel). Fraction numbers and relative molecular weights (Mr) are noted. PKM2 tetramer (fractions 1–10), dimer (fractions 11–14), and monomer (fractions 15–22) were quantified using ImageJ software. The expression of CIP2A and PP2A-Aα/β was tested by western blotting (left panel). (**b**) A549 cells stably expressing *CIP2A* shRNA were transfected with siRNA targeting B56α or control for 36 hours as shown in Fig. 4h, and then the OCR and relative parameters were analyzed. Data represents the average of three independent experiments (mean ± SD). * $P < 0.05$, ** $P < 0.01$, *** $P < 0.001$. (**c**) Representative bright-field images of cell culture media color following B56α depletion in A549 cells stably expressing *CIP2A* shRNA.

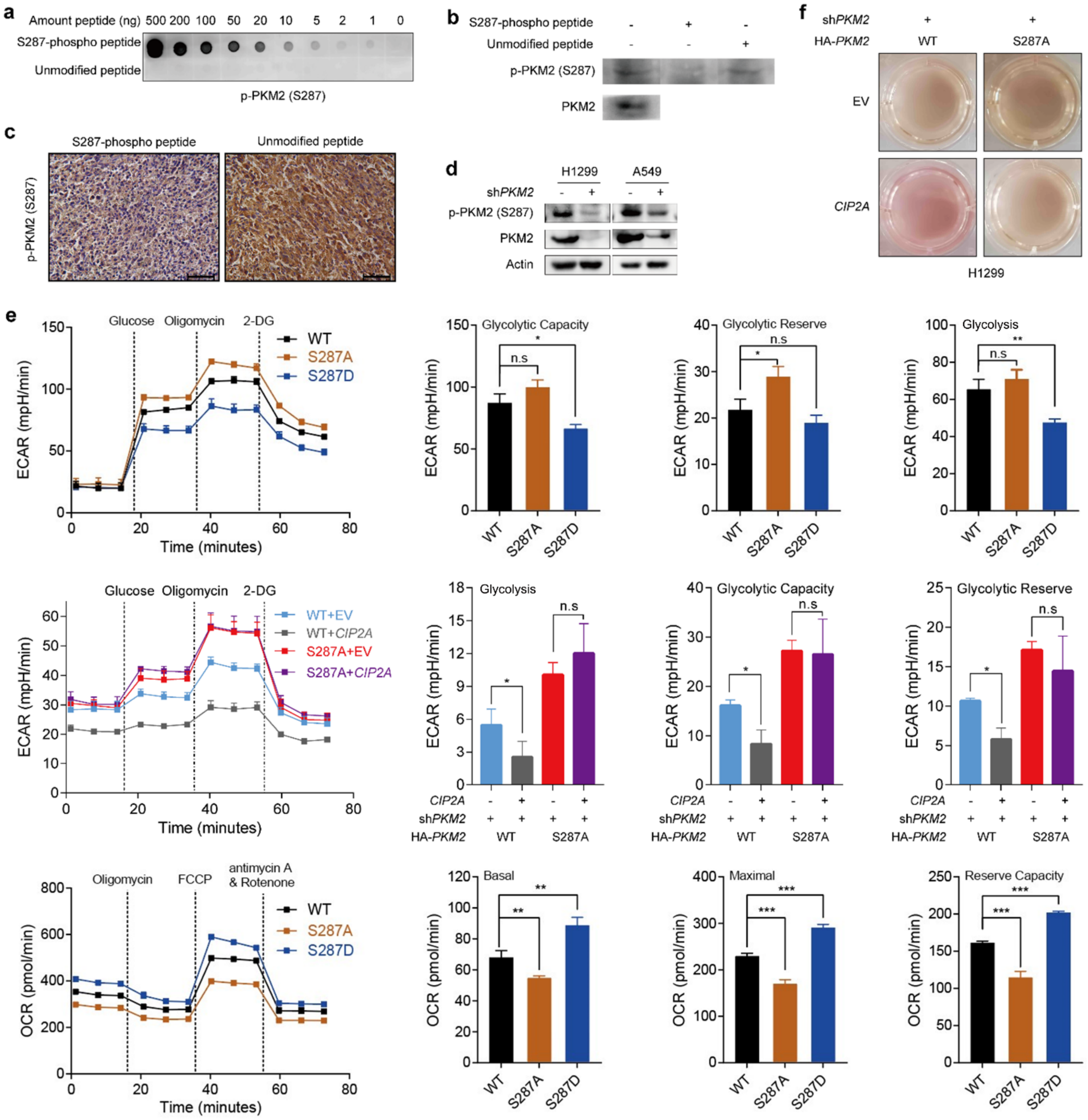

Supplementary Fig. S5. PKM2 serine 287 is a direct dephosphorylation target of B56α. (a) Specificity of the phospho-S287 antibody was characterized by dot blot assay. The nitrocellulose membrane was spotted with S287-phosphopeptide (ILEAS(p)DGI) or unmodified peptide (ILEASDGI) at the indicated amounts and determined by phospho-S287 antibody. (**b, c)** WB specificity of the phospho-S287 antibody was characterized by an immunizing peptide blocking experiment. The phos-S287 antibody was first neutralized with S287-phosphopeptide (ILEAS(p)DGI) or unmodified peptide (ILEASDGI) overnight and then validated by immunoblot (b) and immunohistochemical (c) analyses. Scale bar, 50 µm. (**d)** Specificity of the antibody against phospho-S287 was determined with a knockdown experiment. Total cell extracts

from scramble and *PKM2* shRNA stably expressed cells were examined using the indicated antibodies. (**e**) ECAR and glycolytic parameters were determined in H1299 cells ectopically expressing HA-PKM2 (WT or S287A) and Flag-CIP2A, as shown in Fig. 5k. Data represent the average of three independent experiments (mean ± SD). WT, wild type. * $P < 0.05$; n.s., not significant for the indicated comparison. (**f**) Representative cell culture media images of endogenous PKM2-depleted H1299 cells cotransfected with Flag-*CIP2A* and shRNA-resistant HA-*PKM2* WT/S287A.

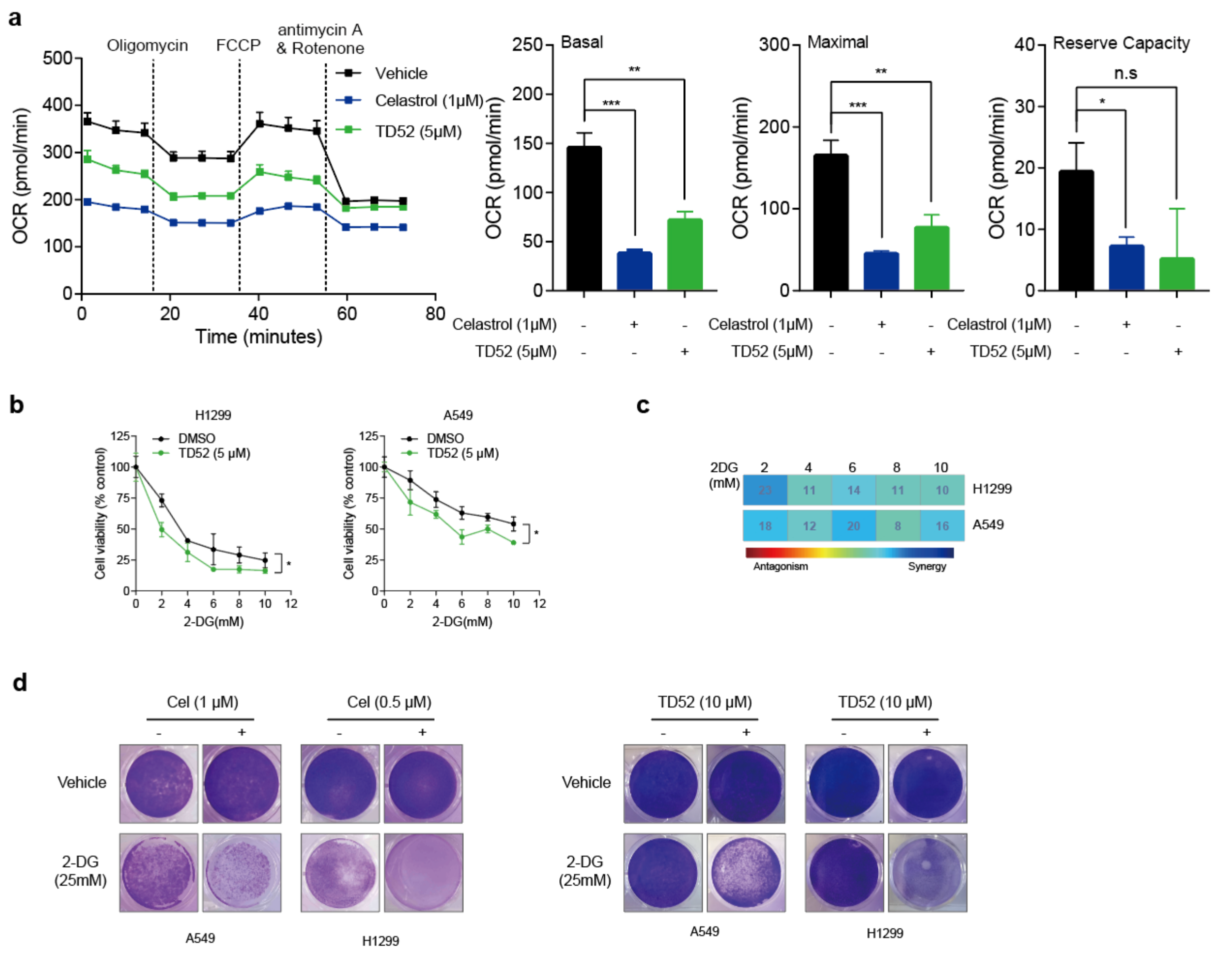

Supplementary Fig. S6. Modulation of CIP2A and PKM2 controls NSCLC cell proliferation. (a) ECAR and glycolytic parameters were determined in A549 cells treated with celastrol (1μM) or TD52 (5μM). * $P < 0.05$, ** $P < 0.01$ for the indicated comparison. (**b, c)** Cell viability of H1299 and A549 cells treated with indicated compounds (b), and the combinatory effects were analyzed by Bliss synergism analysis (c). Two-way ANOVA, * $P < 0.05$, ** $P < 0.01$. (**d)** Crystal violet staining of A549 and H1299 cells after seeding in 12-well plates and treatment with the indicated compounds for 48 h.
